# Transcriptional Activity of Genes Related to the Biotransformation Process in the Development of Colorectal Cancer

**DOI:** 10.3390/ijms262412116

**Published:** 2025-12-16

**Authors:** Grażyna Janikowska, Tomasz Janikowski, Aleksandra Kuźbińska, Mieszko Opiłka, Urszula Mazurek, Zbigniew Lorenc

**Affiliations:** 1 Department of Analytical Chemistry, Faculty of Pharmaceutical Sciences in Sosnowiec, Medical University of Silesia, Jagiellońska 4 Street, 41-200 Sosnowiec, Poland; 2 Silesian College of Medicine in Katowice, Mickiewicza 29 Street, 40-085 Katowice, Poland; 3 Department of Pathomorphology and Molecular Diagnostics, Faculty of Medical Science in Katowice, Medical University of Silesia, Medyków 18 Street, 40-752 Katowice, Poland; akuzbinska@sum.edu.pl; 4 Centrum Medyczne NAVIGARE, Konopnickiej 78 Street, 41-300 Dąbrowa Górnicza, Poland; 5 Faculty of Pharmaceutical Sciences in Sosnowiec, The Karol Godula Upper Silesian Academy of Entrepreneurship in Chorzów, Racławicka 23 Street, 41-506 Chorzów, Poland; umazurek10@gmail.com; 6 Department of General, Colorectal and Polytrauma Surgery, Faculty of Health Sciences in Katowice, Medical University of Silesia, 40-055 Katowice, Poland; zlorenc@sum.edu.pl; 7 Clinical Department of General, Colorectal, and Polytrauma Surgery in St Barbara Regional Specialist Hospital No 5, Medyków1 Square, 41-200 Sosnowiec, Poland

**Keywords:** large colon cancer, *adenocarcinoma*, detoxification phases, gene expression

## Abstract

Colorectal cancer (CRC) remains the third leading cause of mortality among cancer patients in developed countries. Each new study in this field can contribute to better detection, diagnosis, and treatment of this disease. Our study aimed to assess transcriptional activity of genes associated with the biotransformation of xenobiotics and endobiotics in all three phases in the CRC *adenocarcinoma*, including correlations between them, as well as the aromatic hydrocarbon receptor (AhR) pathways. Based on transcriptome analysis (1252 mRNAs) of the CRC tissue and healthy colon, the upregulation or downregulation of 46 significant mRNAs was presented. The study also revealed the downregulation of *AKR7A2* and upregulation of *SLC5A6* and *SLC29A2*, previously undistinguished and potentially therapeutically valuable in CRC. The diagnostic potential of *ADH1C*, *GGT5*, *NQO2*, and *SLC25A5* was demonstrated. It was stated that the *AHR*, *EPHX1*, *GSTP1*, and *SLC25A32* did not correlate in healthy intestinal tissue whereas *AHCY*, *ALDH1A1*, *NNMT*, *GSTM4*, *UGT2B17*, and *SLCO1B3* did not correlate in CRC. The disturbed transcriptional activity of genes related to the biotransformation process at all stages of CRC suggests that this may be the cause of its occurrence; the genes ought to be taken into account in preventive strategies and the treatment of patients.

## 1. Introduction

Despite many healthcare efforts to modify treatment methods and improve prevention, colorectal cancer remains the third most common cancer worldwide and the second leading cause of cancer death [1]. Although endoscopic screening, promoting a healthy lifestyle, and increased awareness of the consequences of environmental pollution (smoking bans in public places and information about its carcinogenic effects) have already led to a reduction in the number of colorectal cancer (CRC) cases in Europe [2], much remains to be performed. It is important to identify new biomarkers and develop affordable diagnostic tests for rapid CRC detection. Many of them demonstrate better sensitivity and specificity than the well-known CEA (Carcinoembryonic antigen) and CA19-9 (Carbohydrate antigen 19-9) [3].

Today, thanks to molecular, genomic, and genetic studies, we know that dysregulation of many genes contributes to the development of CRC, such as the *APC* gene, which leads to the activation of the *Wnt*/β-catenin pathway and contributes to the initiation of the neoplastic process; *TP53* and *PIK3CA* pathways, which contribute to the progression of CRC; and the *SMAD4* and *TGF-β* pathways, whose involvement in metastasis has also been described [4]. Increased gene expression contributes to disease progression, while its suppression also affects the dysregulation of cellular processes, which also influences the treatment process. Therefore, every study in CRC is important and can inspire researchers and practitioners to the combination treatment.

Despite significant progress in the treatment of CRC, surgical removal of the tumor remains the mainstay of treatment. Accurate removal and clinical description require the assessment of various factors, such as tumor location, its absence or presence, the size and local status of local lymph nodes, and distant metastases, according to the global TNM (Tumor Node Metastasis) classification, which defines the clinical stage of cancer in four stages [5]. Tissue differentiation is described using histological classification. Imaging techniques also play a significant role in determining CRC staging and treatment progress. The lower the clinical stage of the disease and the higher the tissue differentiation, the better the patient’s prognosis. CRC treatment strategies depend on all of these factors. In the early stages of the disease and with a low histological grade of the tumor, surgical methods, chemotherapy (typical cytostatic treatment: 5-fluorouracil, leucovorin, oxaliplatin, and irinotecan), and radiotherapy (in rectal cancer to shrink the tumor) are used, which are effective in most of the treated patients, as shown by long-term survival rates [6].

However, a still large number of CRC cases are detected at an advanced stage [7], significantly reducing the chances of effective treatment with typical chemotherapy for CRC. Targeted therapy, immunotherapy, and personalized treatment are used in the advanced stages of CRC, often in different combinations (adjuvant or neoadjuvant therapy) to manage systemic spread and shrink tumors. Surgery is also used to remove the tumor after it has been reduced with chemotherapy and/or radiotherapy and to relieve symptoms. The specific treatment strategy depends on the individual’s cancer and aims to improve quality of life and extend survival, similar to targeted therapy. Molecular research based on transcriptomic and/or genetic studies has contributed to the development of targeted therapies for colon cancer, such as those limiting angiogenesis through acting on vascular endothelial growth factor (anti-*VEGF*), targeting *EGFR* (Epidermal Growth Factor Receptor) and RAS (rat sarcoma) mutations, as well as *KRAS* (Kirsten RAS), *NRAS* (neuroblastoma RAS), *BRAF* (B-Raf proto-oncogene, serine/threonine kinase), *MEK* (mitogen-activated protein kinase kinase), and *TNFA* (Tumor Necrosis Factor A) inhibitor therapies. In response to anti-*EGFR* therapy resistance, *HER2* (Human Epidermal Growth Factor *Receptor*) therapy was developed (amplified and/or overexpressed *ERBB2*/*HER2* triggered constant signals for cell division and survival, driving cancer progression) [8]. Anti-*HER2* therapies demonstrate a non-toxic profile and high efficacy in cases of chemotherapy resistance [9]. Treatment of metastatic colorectal cancer also involves blocking proteins that prevent the immune system from attacking cancer cells (immune checkpoint inhibitors) and *HER2*-specific or CAR T cells (Chimeric Antigen Receptor T cells) reactive to the tumor [10]. There are many more gene therapies targeting specific types of colon cancer, and new ones are constantly emerging, which are in various stages of clinical trials. Most of the genes mentioned above have also been identified in liquid biopsy samples [8,10,11], which may offer hope for improved treatment. Furthermore, there are many natural and synthetic compounds in preclinical studies that show activity against colon cancer cells [12,13].

The highest incidence of CRC in Poland is reported in the Silesian region (a highly industrialized region) [7]. Many global statistical studies link morbidity and mortality to environmental factors such as air, water, and soil pollution. The impact of environmental pollutants is directly proportional to the body’s detoxification capacity and indirectly influences the potential development of cancer [14]. The large intestine, as the final section of the digestive system, is highly folded and has an increased surface area for the absorption of minerals and hydrophilic substances from food residues. This makes it more susceptible to the effects of metabolized xenobiotics. Defense against them involves a three-phase biotransformation system that converts xenobiotics into compounds that facilitate their excretion (oxidation or hydroxylation, etc., and conjugation), but they are sometimes converted into more toxic compounds (e.g., the carcinogenic benzo(a)pyrene (BaP))—produced by various factories, car exhaust, tobacco smoke, burning coal or charcoal—and then metabolized into carcinogenic dihydroderivatives in the first phase of detoxification/biotransformation [15]. All proteins in the three phases of biotransformation are encoded by their corresponding genes. Phase I biotransformation genes encode enzymes, cytochrome P450 monooxygenases (CYPs), which are responsible for converting compounds into compounds with a more hydrophilic structure, facilitating their excretion from the body. The non-CYP group of oxidative enzymes also includes flavin-containing monooxygenases (FMOs), monoamine oxidases (MAOs), peroxidases, xanthine oxidases (XOs), aldehyde oxidase (AO), alcohol dehydrogenase (ADH), and aldehyde dehydrogenase (ALDH). Phase II genes encode enzymes such as glutathione S-transferase (GST), UDP glucuronosyltransferase (UGT), and others that bind phase I products by adding large side groups (glutathione, glucuronosyl, and others), increasing their hydrophilicity and facilitating their excretion from the body. Phase III genes encode transporters, such as the adenosine triphosphate-binding cassette (ABC), and other transmembrane transporters that actively uptake and/or pump conjugated compounds or phase II metabolites across the cytoplasmic membrane [16,17]. Phase III genes also encode solute carrier transporters (SLCs), which, in the form of proteins, facilitate the passage of specific solutes across the membrane and actively transport them against the electrochemical gradient, combining with ions or another solute [16]. This entire three-stage system is called biotransformation (not only of exogenous compounds, such as various chemicals, including pesticides, drugs, environmental pollutants, and food additives, but also of endogenous compounds, such as neurotransmitters, hormones, cellular components, and other synthetic or natural compounds) [14,15,16]. It is known that these enzymes also participate in the metabolism of endogenous substances, transforming them in biochemical pathways and detoxifying and removing exogenous substances (e.g., BaP or TCDD (2,3,7,8-tetrachlorodibenzo-p-dioxin)) from the body that have entered cells through slow accumulation or poisoning [14,15,18]. New roles for genes and proteins involved in biotransformation in health and disease are also being discovered. Biotransformation-related genes are expressed in greater or lesser amounts of mRNA in every cell of the body, although they are specific to certain tissues (e.g., some are found only in the liver, and others in the intestine) [16,19]. Imbalances within and between phases can have serious consequences for the organism. Changes at the mRNA level (transcriptional activity) can contribute to pathological processes, such as the development of disease in the tissue or cellular microenvironment [20].

Among the various theories explaining the causes of cancer, one focuses on an imbalance between the successive phases of xenobiotic/endobiotic detoxification/biotransformation. This may be due to external factors, such as alcohol consumption, smoking, exposure to carcinogenic environmental factors, and the consumption of large amounts of grilled or roasted meat, which are transformed in cells to soluble carcinogenic substances [21,22,23]. Studying changes in the expression of genes associated with biotransformation processes in tumor-affected colon segments at different clinical stages, compared to macroscopically and histopathologically unaffected colon tissue, could help explain these changes and identify potential diagnostic markers and/or therapeutic targets at the transcriptional level. Therefore, the study aimed to analyze the transcriptional activity of genes encoding enzymes and molecules involved in the biotransformation process in stages I–III of colorectal cancer compared with histopathologically normal colorectal tissue and to investigate the correlation between them as well as the AhR receptor pathways.

## 2. Results

### 2.1. Histopathological Confirmation

**Figure 1:**
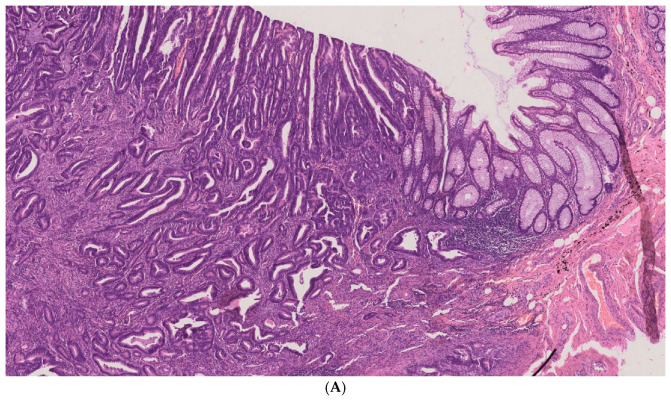

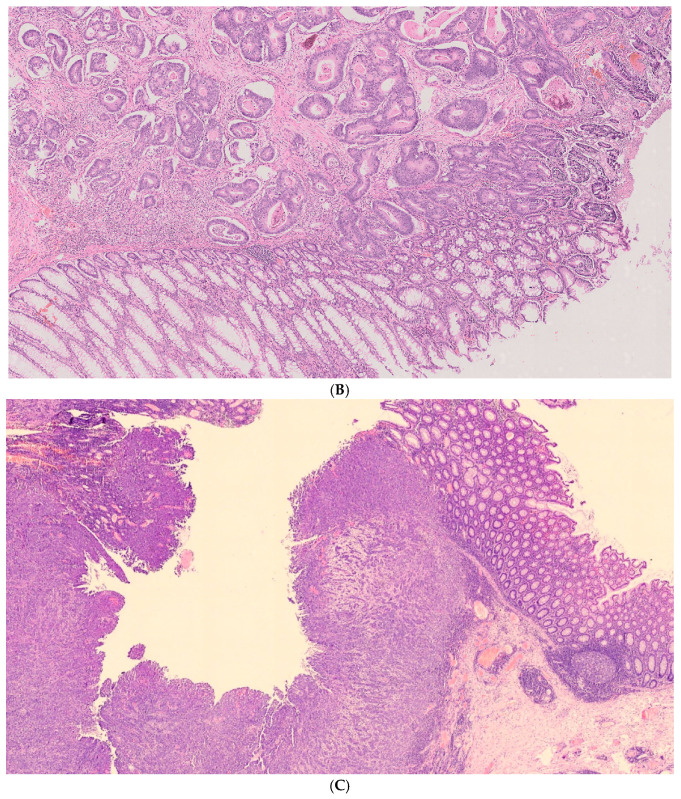
(**A**) Histopathological image of colorectal *adenocarcinoma* G1 (on the left, cancerous tubules infiltrating the *submucosa* and *muscularis propria*, and on the right, a normal colonic wall) (H&E, ×4). (**B**) Histopathological image of colorectal *adenocarcinoma* G2 (primitive, tubules of various shapes forming solid and cribriform groups) contrasting with the normal mucosa below (H&E, ×4). (**C**) Histopathological image of colorectal *adenocarcinoma* G3 (on the left, cancer without glandular differentiation) and normal colon mucosa (on the right, a normal colonic wall) (H&E, ×4).

The results of the histopathological images confirm the colon tumor as *adenocarcinoma* of colorectal cancer in different grades, G1, G2, and G3, at 4× magnification (Figure 1). Histopathological grading is based on the amount of glandular differentiation in the tumor. Well-differentiated (G1) *adenocarcinoma* has more than 95% glandular tissue, moderately differentiated (G2) has 50–95%, and poorly differentiated (G3) has less than 5%. Figure 1A–C show the difference between large colon cancer (CRC) and healthy control colon tissue (CC) in the histopathological image, which is also visible to a surgeon who excises the cancer with a wide margin and selects material for investigation. The figures show histopathological images of CRC with the unchanged colon tissues. More images of this tumor in different grades of differentiation, G1, G2, and G3, can be seen in the Supplementary Materials in Figures S1–S3.

The images are examples used to demonstrate the contrast between cancerous and non-cancerous tissue.

### 2.2. Quality Confirmation

**Figure 2:**
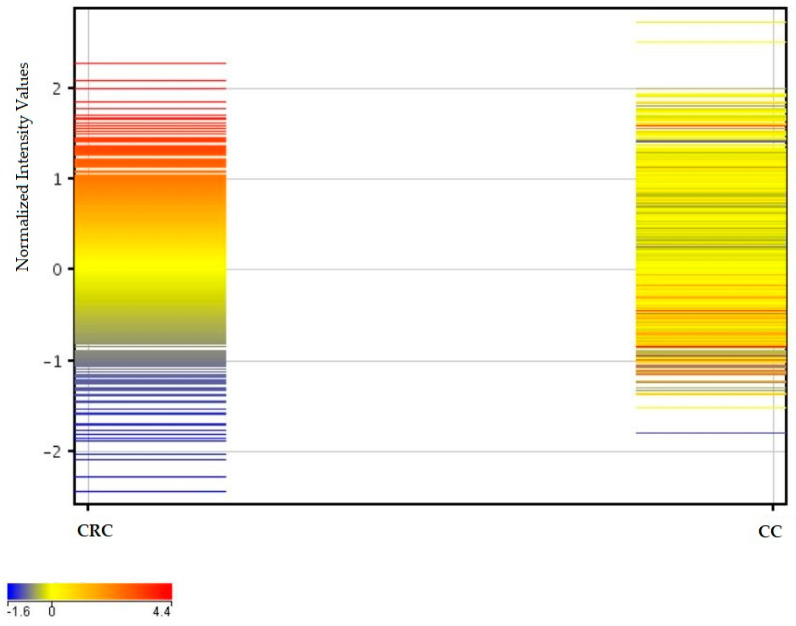
Comparison of the mean fluorescence intensity values of mRNAs for CRC and CC samples. Explanation: CRC—examined colorectal cancer tissues; CC—healthy colon tissue samples. Legend: blue to yellow colors represent lower values, and yellow to red represent higher values.

Quality control analysis of the microarrays is shown in Figure S4 (before removing one). All analyses were performed with exclusion and inclusion criteria for samples. Differences between CRC and CC are visible in the different expressions of the same mRNA from all examined samples in Figure 2. Magnitude segregation of the mRNA mean fluorescence intensity signals in the form of colors from cold (blue—lowest fluorescence) to warm (red—highest fluorescence) for CRC and CC samples is presented as a profile plot.

**Figure 3:**
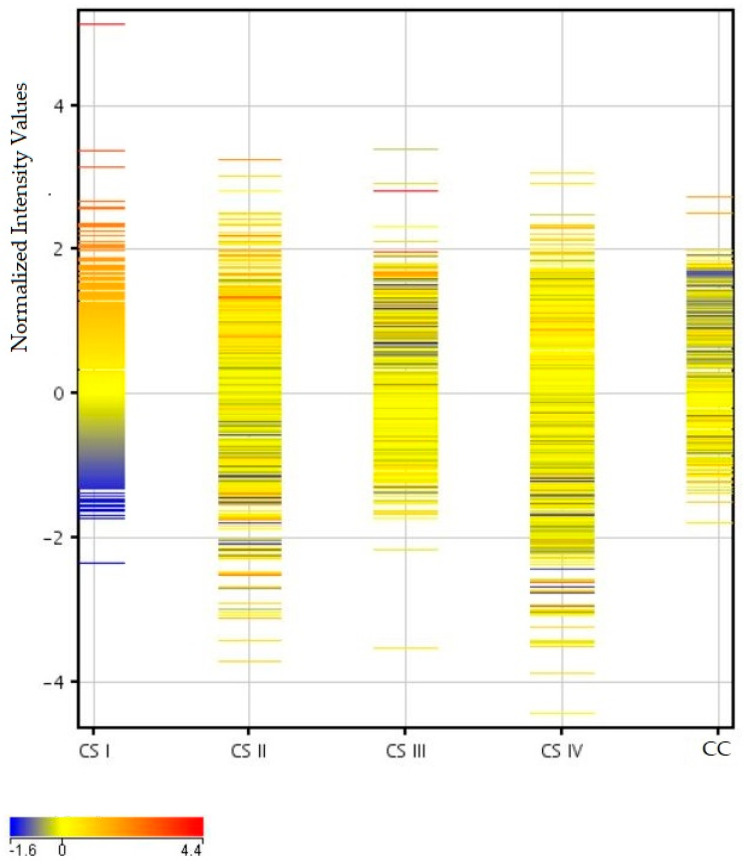
Comparison of the mean fluorescence intensity values of mRNAs in CRC in particular clinical stages (CS I—IV) and control (CC) samples. Legend: blue to yellow colors are lower values, and yellow to red are higher values.

The next profile plot in Figure 3 shows the differentiation of mRNA fluorescence signals in individual clinical stages of CRC (CSI—CSIV) and healthy colon tissue (CC). The mRNA fluorescence profile of the first clinical stage (CSI) significantly differs from the others, and it is dominated by an increase in fluorescence intensity for a large number of transcripts. Furthermore, a difference is visible between the subsequent CRC stages and the CC.

### 2.3. Similarities and Differences in Fluorescence Signals of mRNAs

**Figure 4:**
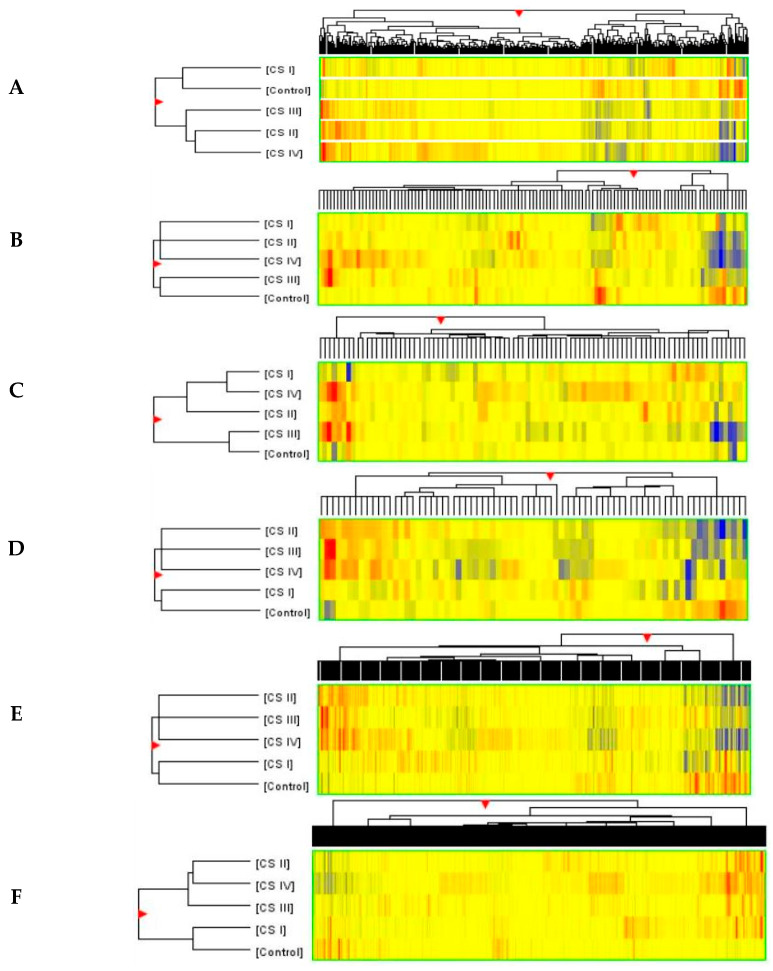
Self-organizing maps for fluorescence intensity of biotransformation process transcripts in the examined CRC of CS I—IV and CC samples. Legend: (**A**) biotransformation 366 mRNA, (**B**) phase I functionalization 121 mRNA, (**C**) cytochrome P450 91 mRNA, (**D**) phase II conjugation 75 mRNA, (**E**) ATP-binding cassette 69 mRNA, (**F**) transporter 456 mRNA (phase I—(**A**–**C**); phase II—(**D**); phase III—(**E**,**F**)). High intensity of fluorescence signals—red; low—blue. The colors of particular bands of transcripts show similarities (the same color) and differences (different colors in different stages but the same mRNAs). CSI—clinical stage I, CSII—clinical stage II, CSIII—clinical stage III, CSIV—clinical stage IV of colorectal cancer development.

To present the similarities and differences between the fluorescence intensity of various mRNAs in individual panels of biotransformation processes (phases I–III), and, therefore, the relative arrangement of the examined CRC in four clinical stages (CSI-IV) and CC, self-organizing maps (SOMs) were performed using the Euclidean distance and Ward methods. These relationships are shown in Figure 4 as particular clusters for both entities on the top (fluorescence signal intensity for particular mRNAs) and conditions on the left (samples). All lists of entities for all biotransformation process panels of mRNA are presented in Tables S5–S10.

The fluorescence signal intensity of individual mRNAs (entities) in terms of the biotransformation process (366 mRNA), phase I functionalization (121 mRNA), cytochrome P450 (91 mRNA of CYP’s), phase II conjugation (75 mRNA), ATP-binding cassette (69 mRNA, phase III), and transporters (456 mRNA, phase III) show similarities and differences in the sample arrangement of visible clusters. The result of significant repetition of some mRNAs (fluorescence signals) for both phase I functionalization and CYPs, and phase II conjugation and ATP-binding cassette panels, is an unambiguous similarity in the distribution of the sample set (condition) in visible clusters. Different arrangements of the samples in the biotransformation process and transporter panels confirm differences in the fluorescence signal intensity of their mRNAs. The control sample’s initiation into each visible cluster instead of the first demonstrates its molecular uniqueness in these comparisons with cancer samples, as illustrated in Figure 4.

### 2.4. Analysis of Variance and Post Hoc Test

Each examined panel of transcripts related to biotransformation processes showed a different number of statistically significant mRNAs, depending on the *p*-value, which are presented in Table 1.

From a total of 366 mRNAs in the general panel of biotransformation, 98 were significant at a *p* of less than 0.05, and 29 were significant in phase I functionalization (total 121). *CYPs* (total 91) had the lowest number of differential mRNAs, with only nine. In phase II conjugation, of a total of 75, 14 were statistically significant. In the phase III ATP-binding cassette panel, from a total of 69, 11 had differential mRNA, and 63 transporters from a total of 456 were significant in these microarrays. The number of significant genes in individual panels depends on the applied level of significance. The lower the *p*-value, the lower the number of statistically significant mRNAs. The ANOVA test results for all panels of mRNAs are shown in Tables S11–S16.

**Figure 5:**
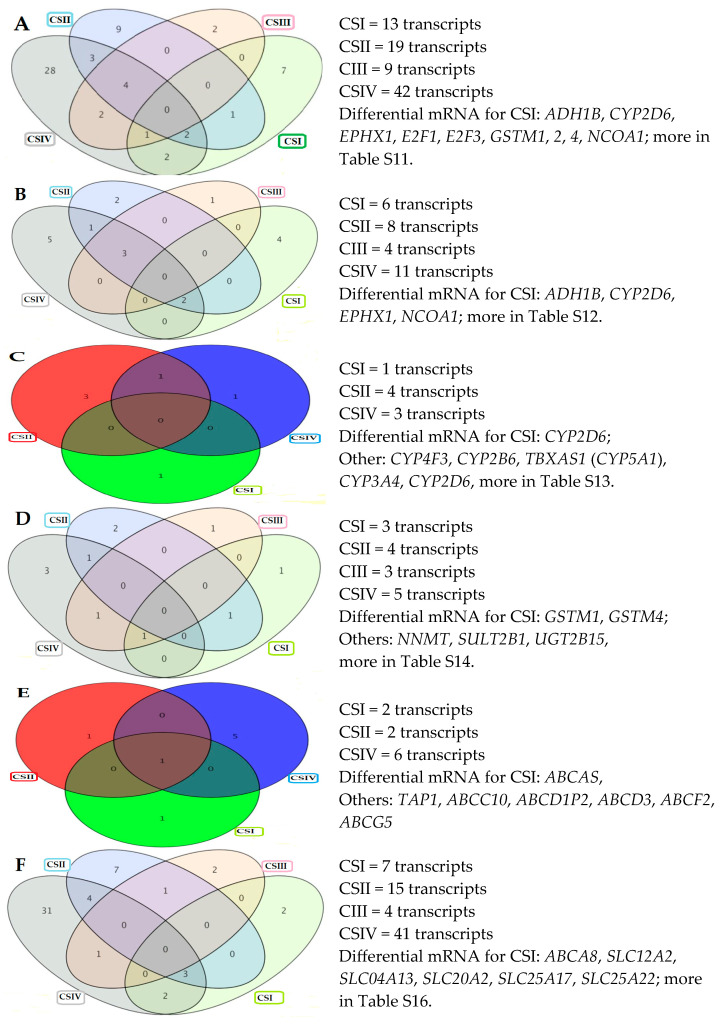
Venn diagrams of biotransformation process panels in Tukey’s HSD *post hoc* test. Legend: (**A**) biotransformation, (**B**) phase I functionalization, (**C**) cytochrome P450, (**D**) phase II conjugation, (**E**) ATP-binding cassette, (**F**) transporters. CSI—clinical stage I, CSII—clinical stage II, CSIII—clinical stage III, CSIV—clinical stage IV of colorectal cancer development. HSD—highly significant difference.

From the next results of the Tukey HSD *post hoc* test (*p* ≤ 0.05), a Venn diagram with numbers of significant mRNA in each comparison (CS vs. CC) was obtained for all panels, as shown in Figure 5A–F. Characteristic mRNAs for the first stage of CRC could be candidates for the group of genes differentiating cancer from healthy intestinal tissue (CSI vs. CC in CRC), which are listed next to the diagram. Unfortunately, not all probes for the assessed gene are expressed. Therefore, those that did not meet the criterion were excluded from the analysis. Additionally, not all probe sets of mRNAs corresponding to a specific gene are statistically significant in the analysis of variance (ANOVA), and only significant transcripts with an FC ≥ 2.0 at least at one clinical stage of the CRC are accepted. Rigorous statistical elimination of transcripts in all analyzed panels (*p*-value, Benjamini–Hochberg correction, fold change) allowed distinguishing the 23 significant genes (29 mRNAs) shown in Table 2 in the first (I) and second (II) phases, and 18 genes (20 mRNAs) are shown in Table 3 in the third (III) phase of the biotransformation process.

### 2.5. Differentially Expressed Genes

Between transcripts linked to the first phase, the *AHCY* (encodes *adenosylhomocysteinase*), *CYP2B7P1* (encodes cytochrome P450 2B7P1), and *DPEP1* (encodes dipeptidase 1) expression increased with cancer growth in subsequent stages of the disease. This is evidence of the involvement of the biotransformation process in the development of CRC. Next, the expression of *ADH1C* (which encodes alcohol dehydrogenase 1C) only increases in CSI compared to the control and then drastically decreases in CSII to CSIV, showing a predisposition to be a good biomarker for CRC. Conversely, *GGT5* (which encodes gammaglutamyl transferase 5) is decreased in CSI and is much higher in subsequent stages.

The next highlighted mRNAs, *ADH5* (encodes alcohol dehydrogenase class III), *AKR1B10* (encodes aldo-keto reductase 1 B10), *AKR7A2* (encodes aldo-keto reductase family 7 member 2), *ALDH1A1* (encodes aldehyde dehydrogenase 1A1), *EPHX1* (encodes microsomal epoxide hydrolase), *EPHX2* (encodes soluble epoxide hydrolase), *MAOA* (encodes monoamine oxidase A), and *PTGS1* (encodes prostaglandin-endoperoxide synthase 1), are silenced at all clinical stages compared to CC (Table 2).

The second phase represented *GSTP1* (encodes glutathione S-transferase π) and *NNMT* (encodes nicotinamide N-methyl transferase) expression, which increased in all stages of cancer development. But, *NQO2* (encodes N-ribosyl dihydronicotinamide: quinone dehydrogenase 2) has reduced expression in CSI compared to CC and then increased it in subsequent stages. *GSTM1*, *2*, *4* (encode glutathione S-transferase Mu 1, 2, 4), *UGDH* (encodes UDP-glucose 6-dehydrogenase), *UGP2* (encodes the enzyme UDP glucose pyrophosphorylase 2), *UGT1A9*, and *UGT2B17* (encode uridine diphosphate glucuronosyl transferase 1A9 and 2B17) are silenced in all clinical stages of CRC (Table 2).

In both the first and second phases of biotransformation, most transcripts are silenced. However, in the third (III) phase of biotransformation, most of the transporters are upregulated, as seen in Table 3.

The transcripts *ABCB2* (encodes ATP-binding cassette subfamily B member 2) and *SLC2A3* (encodes solute carrier 2A3), and ten more solute carriers representing the third phase of biotransformation (Table 3), are upregulated in subsequent stages, but *ABCA8* (encodes ATP-binding cassette subfamily A member 8), *ABCD3* (encodes ATP-binding cassette subfamily D member 3), and *ABCG2* (encodes ATP-binding cassette subfamily G member 2) are downregulated. Differential expressions in the initial stage of cancer (CSI or CSI and CSII), such as for *ADH1C*, *GGT5*, *NQO2*, and *SLC25A5* (Table 2 and Table 3, bolded), may indicate them as candidates for diagnostic biomarkers.

Many molecules constituting canonical and non-canonical cellular pathways are closely associated with the AhR receptor. Seventy-three probes of mRNA (Table S17) related to the AhR were identified. All of them met the criteria and are shown in Table 4. Their expression is uniform and indicates their involvement in the biotransformation process of CRC, but the FC value does not exceed 2.

All statistically significant mRNAs for the AHR pathways are shown in Table S18. They are also found in the panel of 366 mRNAs involved in the biotransformation process. The *AHR* (aryl hydrocarbon receptor), *CPB2* (encodes carboxypeptidase B2), *E2F1* (encodes a transcription factor crucial for cell cycle regulation, DNA repair, and apoptosis), *E2F6* (encodes a transcription factor that controls the cell cycle), and *PRB3* (encodes proline-rich protein BstNI subfamily 3) do not meet the criteria, but all of them have enhanced expression. Transcripts of *PTGS1* (this group of molecules also acts with the AhR) have significantly diminished expression and are presented in Table 2. A statistically selected set of significant genes associated with AhR pathways confirms its regulatory role in the development of CRC. More information about selected significant genes is described in Table S19. No differences were observed between the obtained results when divided by age and sex.

### 2.6. Correlation Analysis

To further assess the transcriptional activity of statistically selected transcripts involved in the biotransformation process (all three phases) and AhR pathways, correlation analysis was performed. The results illustrating their relationships in a normal colon (CC), in the comparison of CC versus *adenocarcinoma* (AC), and in AC vs. AC are presented as statistically significant correlation coefficients (r) and probability values (*p* < 0.05) in Table 5, Table 6 and Table 7 for all three phases of biotransformation. The highest number of significant correlations between the fourteen significant mRNAs (including *AHR*) selected in the ANOVA test was found in CC samples in phase I (34 correlations, including 19 positive and 15 negative out of 91 total), and the lowest was found in CC vs. AC (nine correlations, including four positive and five negative out of 196 total). Fourteen were found in CRC tissue AC vs. AC (eight positive and five negative), which are shown in Table 5 (total correlations of the comparison are shown in Tables S20–S22, significant and insignificant). In the comparison CC vs, CC, *CYP2B7P1* correlated with eight genes, indicating its important role in xenobiotic detoxification in these cells.

Similar to phase I, the highest number of phase II genes correlated in control colon tissue (16 correlations for eleven transcripts, including seven negative ones), as shown in Table 6. Furthermore, a lower number of correlations was found for CC vs. AC (nine correlations, including two negative), and there were even fewer in AC vs. AC (four significant correlations and two negative correlations). All correlations (significant and insignificant) from phase II are shown in Tables S23–S25.

In phase III, 72 statistically significant correlations (48 positive and 24 negative) were found for CC vs. CC, eighteen (twelve positive and six negative) for CC vs. AC, and forty (28 positive and 12 negative) for AC vs. AC (Table 7). All correlations (significant and insignificant) from phase III are shown in Table S26–S28.

No statistically significant correlation with the *AHR*, *EPHX1*, *GSTP1*, and *SLC25A32* was found in CC vs. CC, which may indicate that it has a role other than biotransformation in the large colon cell. These mRNAs correlate with others in CRC (AC vs. AC).

The *AHR* negatively correlates with *ADH1C* and *AKRB10* in CC vs. AC and with *DEPEP1* in AC vs. AC. *EPHX1* positively correlates with *ADH5* and *GGT5* and negatively correlates with *MAOA* in CC vs. AC. It positively correlates with *GGT5*, *AKR7A2*, and *PTGS1* in CRC. The phase II gene *GSTP1* negatively correlates with NQO2 in CC vs. AC and with *UGP2* in CRC. *SLC25A32* only positively correlates with *SLC5A1*, *SLC6A14*, and *SLC25A15* in CRC.

A lack of significant relationships with *AHCY*, *ALDH1A1*, *NNMT*, *GSTM4*, *UGT2B17*, and *SLCO1B3* was found in CRC (AC vs. AC), which may indicate no cooperative role of these genes in CRC. These mRNAs correlate with others in the healthy colon (CC vs. CC) or CC vs. AC (*AHCY*, *NNMT*, *GSTM4*, *UGT2B17*).

In each phase of biotransformation, most relationships between genes occur in the healthy large intestine (CC vs. CC). Correlations between phases I vs. II, II vs. III, and I vs. III for CC and AC were also examined and are presented in Tables S29–S34, respectively. All the correlations shown indicate high transcriptional activity of the studied molecules and the many dependencies between them.

## 3. Discussion

Transcriptional activity of genes related to the biotransformation process in the healthy large colon and in CRC tissues was assessed in three phases, and additionally, it was connected with aryl hydrocarbon receptor canonical and non-canonical pathways.

The analysis allowed for the identification of mRNAs with diagnostic predispositions: *ADH1C*, *GGT5*, *NQO2*, and *SLC25A5*. Moreover, all statistically significant mRNAs in all three phases of xenobiotic/endobiotic biotransformation were identified. Mutual relationships of all significant genes in the healthy colon tissue and CRC were determined. The involvement of the AHR and its associated genes in CRC was confirmed, demonstrating its likely role through *E2F1* in this cancer.

The study indicated that phase I transcripts, *ADH1C* and *GGT5*, phase II *NQO2*, and phase III *SLC25A5* may have diagnostic and therapeutic potential in CRC due to their differential expression in the early phase of this cancer. *ADH1C* expression was only upregulated in the first clinical stage of CRC, but in CSII-CSIV, it was downregulated, which can indicate that this gene is a good diagnostic biomarker. A lower expression of *ADH1C* mRNA was found in intestinal epithelial cancer tissues compared to healthy tissues [24], which was only confirmed in the present work for CII-IV. Another study stated that decreased expression of *ADH1C* from *adenoma* to early and advanced stages of colorectal carcinomas was associated with reduced all-trans retinoic acid biosynthesis. Hence, it led to alterations in cell growth and differentiation in the colon and rectum, contributing to cancer progression [25]. The results show that *ADH1C* is the only phase I transcript that correlates with the *AHR* in CC vs. AC (Table 5; negatively).

Next, *GGT5* was distinguished as a candidate for diagnostic molecules. *GGT5* hydrolyzes the gamma-glutamyl moiety with the capacity to cleave the gamma-glutamyl moiety of glutathione [26]. Increased expression of *GGT5* mRNA was found for breast, gastric, and colorectal cancer tumors in comparison to normal tissues [27]. In our study, *GGT5* expression was increased with the clinical stage CSII-IV, but it was silenced in the CSI of the CRC tissues, which can indicate that this gene can be a good diagnostic marker. Furthermore, this transcript is active in correlations in both healthy colon tissue and CRC.

*NQO2* was overexpressed in CRC tissue samples, except for CSI, where FC was slightly decreased. Chen et al. showed downregulated expression of *NQO2* in CRC tissues from patients who already had liver metastasis [28]. Moreover, its expression can protect against quinone-induced skin carcinogenesis and stabilize p53 in the cell [29]. NQO2, acting as a detoxification enzyme, catalyzes the reduction in electrophilic estrogen quinones (estradiol) [30], revealing a relationship between breast cancer [31]. Furthermore, this transcript is active in correlations in both tissues and correlates negatively with *UGT1A9* in CRC.

*SLC25A5* in CSI-II was upregulated in CSI-II and downregulated in CSIII-IV in CRC, having diagnostic potential for this disease. A study showed that high expression of *SLC25A5* indicated a longer survival time of patients with colon cancer [32]. This gene is responsible for nucleotide transport, mediates the exchange between ADP in the cytoplasm and ATP in the mitochondria [33], and correlates with glucose accumulation in various types of cancer [34], which is why different expression levels in different cancers and actions through different signaling pathways are observed [33,34]. There are many uncertainties associated with it.

The research shows selected transcripts (bolded in Table 2 and Table 3) as candidates for further investigation and observation. This transcript is active in correlations with both healthy colon tissue and CRC.

Among the significant genes with increased expression in CRC was *AHCY*, encoding adenosylhomocysteinase. *AHCY* expression was upregulated in colon cancer compared with uninvolved colon mucosa [35]. Studies using mice with Apc deletion demonstrated that pharmacological inhibition of *AHCY* reduced its intestinal tumor burden, demonstrating the potential of this gene in CRC treatment [36]. The obtained results support the possibility of using the upregulated *AHCY* in all CS in the treatment of CRC. This transcript is not involved in any relationships with other genes in CRC; it is only stimulated by *DPEP1* and negatively stimulates *ADH5* in CC vs. AC (Table 5).

Next, with increased expression in CRC, *CYP2B7P1*, which encodes a metabolic enzyme, allowed the cell to obtain energy from lipid metabolism and to alter the cell membrane composition. It plays a role in cell proliferation, survival, and cancer cell metastasis. Deregulation of this process was especially observed in different neoplasia. *CYP2B7P1* showed potential as a prognostic factor in lung *adenocarcinoma* [37]. On the other hand, *CYP2B7P1/2B6* was correlated with oxidative stress in clinical trauma indices, specifically blunt trauma [38]. *CYP2B7P1* has been identified in colorectal cancer as an early differentially expressed gene that persists until tumor formation [39]. The presented results showed that *CYP2B7P1/2B6* was upregulated in all CRC clinical stages in comparison to the control; the highest fold change was noted for CSIV, which confirmed its involvement in the development of this tumor.

**Figure 6:**
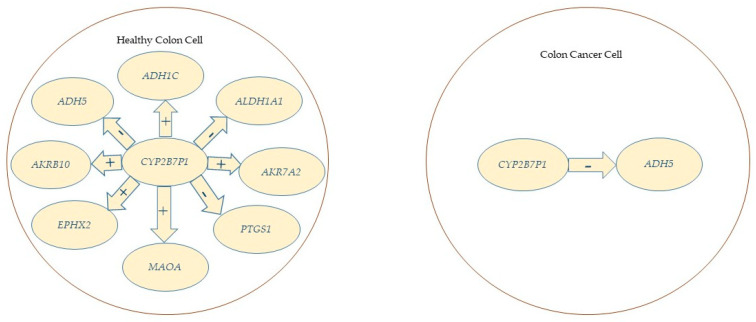
Relationships between *CYP2B7P1* and other genes in healthy large colon tissue and CRC (Table 5). Legend: +, positive correlation; −, negative correlation.

Although *CYP2B7P1* shows the highest activity in relationships with eight genes in healthy colon tissue, as shown in Figure 6, only a negative relationship with *ADH5* was maintained in CRC.

DPEP1 hydrolyzes dipeptides and metabolizes leukotriene, and it is a marker for the transition from low-grade to high-grade intraepithelial neoplasia. Like our results, the expression of *DPEP1* was increased in human CRC tissue samples compared with normal mucosa and was indicated as an adverse prognostic factor in CRC [40]. Due to the increased expression of this gene, it may be used for further studies to silence CRC. *DPEP1* correlates positively with *GGT5* in healthy intestinal tissue and with *AHCY* in CC vs. AC. However, it negatively correlates with the *AHR* and *ADH5* in CRC.

Next, distinguished transcripts in phase I (*ADH5*, *AKRB10*, *AKR7A2*, *ALDH1A1*, *EPHX1*, *EPHX2*, *MAOA*, and *PTGS1*) have decreased expression in all stages of CRC development.

*ADH5* has a low expression in the CSI-IV of CRC, and it is the lowest in CSIV. This gene encodes alcohol dehydrogenase class III, which metabolizes long-chain alcohols and omega-hydroxy fatty acids, eliminates formaldehyde (protecting cells), oxidizes S-(hydroxymethyl)glutathione, forms S-formylglutathione, and regulates nitric oxide in cells [41]. A comparison of class III ADH isoenzyme levels in biopsy specimens of healthy colon mucosa and cancer tissues revealed slight differences in their activity. Similar results were obtained in samples from alcohol drinkers and nondrinkers [42]. The expression of *ADH5* was lower in CRC than in healthy colon tissues. The transcript shows high activity in the healthy colon (five negative correlations with *CYP2B7P1*, *ADH1C*, *GGT5*, *AKRB10*, and *EPHX2* and two positive correlations with *ALDH1A1* and *PTGS1*), and in CRC, it correlates negatively with *DPEP1* and *CYP2B7P1* and positively with *MAOA* (Table 5).

*AKR1B10* was downregulated in CRC tissues compared to the adjacent normal colorectal tissues [43], which is similar to the presented results. *AKR1B10* encodes aldo-keto-reductase, an enzyme that takes part in the metabolism of retinoids, aliphatic aldehydes, and ketones [44]; it participates in the regulation of fatty acid biosynthesis via the ubiquitination–proteasome pathway [45]. *AKRB10* correlates positively with *CYP2B7P1*, *ADH1C*, *EPHX2*, *MAOA* and negatively with *ADH5*, *ALDH1A1*, and *PTGS1* in CC and with the *AHR* (CC vs. AC). It also correlates positively with *ADH1C* and *MAOA* and negatively with *GGT5* in CRC.

*AKR7A2* encodes an enzyme that belongs to the AKR superfamily, previously AKR1B10, too. Next, with decreased expression in phase I, AKR7A2 is involved in the metabolism of acrolein, aflatoxin, and many other substances [36]. In humans, its presence was found in the upper regions of the intestine [46] and in neuroblastoma [47]. The role of *AKR7A2* in the pathogenesis of CRC is unknown. *AKR7A2* correlates positively with *AHCY*, *CYP2B7P1*, and *EPHX2* in the healthy colon and in CRC with *EPHX1*, which correlates only in cancerous tissue (Table 5).

ALDH1A1 has multiple functions in the cell, starting with the decomposition of aldehydes to carboxylic acids and ending with it taking part in the biosynthesis of all-trans-retinoic acids. Its gene expression was correlated with cancer stem cell recognition, but overexpression is associated with poor cancer prognosis [48]. High levels of *ALDH1A1* were associated with a poorly differentiated histology. The expression patterns of ALDH1A1 were heterogeneous in CRC and corresponding adjacent tissues [49]. Decreased expression of *ADH1A1* in our study and a lack of relationships with other transcripts in CRC may be a good prognosis for patients.

*EPHX1* encodes microsomal epoxide hydrolase that plays a role in the detoxification of epoxides (products of various metabolic processes of endogenous and xenobiotic substances) to convert them to less toxic trans dihydrodiols. Expressions of *EPHX1* and *EPHX2* were significantly decreased in CRC and presented as diagnostic and prognostic biomarkers for colorectal cancer [50]. *EPHX1* in our findings was also decreased and showed only a positive correlation with other transcripts (*GGT5*, *AKR7A2*, and *PTGS1*). Lack of correlations with other transcripts in the healthy large intestine may indicate its limited role in this tissue. However, in CC vs. AC, a positive correlation with *ADH5* was found (Table 5).

*EPHX2* encodes the soluble epoxide hydrolase that plays a role in lipid metabolism (breakdown of epoxyeicosatrienoic acids), which takes part in anti-inflammatory and cytoprotective processes. The dysregulation of *EPHX2* was connected to hypertension, hypercholesterolemia, cardiovascular diseases, and hepatocellular carcinoma [51]. Decreased expression of this transcript in CRC and high activity in nine correlations (positive with *CYP2B7P1*, *ADH1C*, *GGT5*, *AKRB10*, *AKR7A2*, and *MAOA* and negative with *ADH5*, *ALDH1A1*, and *PTGS1*) in healthy intestines that were only positive with *ADH1C* in CRC were found (Table 5).

The next silenced gene in CRC is *MAOA*, which encodes monoamine oxidase. Its role is not well understood in CRC pathogenesis, but continuous downregulation of its expression was stated [52], and it is suggested to be a biomarker for progression [53], similar to the presented findings. Dysfunction of oxidative deamination of amines is associated with progressive gene silencing and can lead to reduced oxidation of norepinephrine, serotonin, dopamine, and tyramine [54,55]. When comparing the results of *MAOA* expression in different cancers, they are inconsistent and differ per malignancy. For example, overexpression was found in classical Hodgkin lymphoma and glioma brain tumors, but downregulation was observed in melanoma, small cell lung carcinoma, malignant pleural mesothelioma, renal carcinoma, breast, hypopharyngeal, gastric, papillary thyroid, colon, and cholangiocarcinomas [53,55]. It is a correlation activity that was observed in both healthy tissue (positive with *CYP2B7P1*, *ADH1C*, *AKRB10*, and *EPHX2* and negative with *PTGS1*), as well as CC vs. AC (positive with *MAOA* and negative with *EPHX1* and *PTGS1*) and CRC (positive with *ADH1C*, *ADH5*, and *AKRB10*, and negative with *GGT5*) (Table 5).

Decreased expression in CRC was also observed; it was significant and met the criteria for *PTGS1* (other names include *COX1*, *PGHS-1*, and *PTGHS*), which encodes prostaglandin-endoperoxide synthase 1, which converts arachidonates to prostaglandines (key mediators in inflammation capable of playing a dual role, pro-inflammatory or anti-inflammatory, depending on context) [56]. This gene was involved in the process of carcinogenesis, where its expression was increased or decreased, as in the breast [57] or bladder [58], respectively. Silenced *PTGS1* expression was observed in colon cancer *adenocarcinoma* [59], as shown in the results. Furthermore, a correlation activity was observed in healthy tissue (positive with *ADH5* and *ALDH1A1* positive and negative with *CYP2B7P1*, *ADH1C*, *AKRB10*, *EPHX2*, and *MAOA*) in comparison to CC vs. AC (only negative with *MAOA*) and CRC (only positive with *EPHX1*) (Table 5).

The phase I biotransformation process in the studied CRC tissue can be characterized by oxidative stress and lipid, glutathione, aldehyde, many toxic compounds, and retinol metabolism. This can be a reason for the enhanced activity of *CYP2B7P1*. The expression of these genes increases in parallel with the clinical stages of CRC. On the other hand, the expression of most genes encoding enzymes involved in the metabolism of the first phase was significantly decreased in all clinical stages.

*GSTP1* and *NNMT* are among the differentially expressed genes taking part in the second phase of biotransformation.

The presented results confirm the increased expression of *GSTP1* in colorectal cancer [60,61], which issimilar for *NNMT* [62,63]. The first one, *GSTP1*, encodes glutathione S-transferase pi 1, which catalyzes reactions between glutathione and lipophilic compounds with electrophilic centers, leading to the neutralization of toxic compounds, xenobiotics, and products of oxidative stress [64]. *GSTP1* only shows correlations with *NQO2* in CC vs. AC and *UGP2* in CRC.

The second one, *NNMT*, encodes the nicotinamide (vitamin B3) N-methyltransferase, which controls the intracellular concentration of nicotinamide, a precursor of NAD^+^ [65]. The expression of *NNMT* in the presented CRC was increased in all clinical stages, with a maximum value of *FC* at 5.5 in CSIII. Overexpressed *NNMT* is characteristic of many cancers [62]. Moreover, the activity of *NNMT* is mostly correlated with inflammation but can regulate multiple metabolic pathways, leading to increased proliferation and metastasis [65]. *NNMT* shows a negative correlation with *UGP2* in CC, a positive correlation with *NQO2*, *GSTM2*, and *UGT2B17* in CC vs. AC, and a lack of relation in CRC.

Among downregulated differentially expressed genes taking part in the second phase of biotransformation, our results confirm their participation in CRC, namely, *GSTM1* [60], *GSTM2* [60,66], *GSTM4* [66], *UGDH* [67], *UGP2* [68], *UGT1A9*, and *UGT2B17*.

Analysis of *GSTM1* and *GSTM2* showed that these genes were related to cell cycle, metabolism, and detoxification processes, as well as the *Wnt* signaling and *NF-κB* pathways [66,69]. *UGDH* mRNA expression levels were significantly lower in CRC tissues than in normal tissues and can potentially serve as a prognostic indicator for this cancer [67], similar to the presented findings.

The last two differentiated genes, *UGT1A9* and *UGT2B17*, in the second biotransformation phase were both downregulated in the tested CRC tissue, which confirms previous works [70,71,72]. Our findings also confirm that *UGT2B17* might be a CRC stage-related gene [72].

These genes encode the uridine 5′-diphospho-glucuronosyltransferase enzyme responsible for transferring glucuronic acid to a variety of substrates, e.g., bilirubin, bile acids, fatty acid derivatives, glucocorticoids, mineralocorticoids, retinoids, steroids, or thyroid hormones. They play a vital role in the detoxification process [71].

Only seven mRNAs exhibit correlation activity in this phase in CRC: two positive, *GSTM1* with *GSTM2* and *UGDH* with *UGP2*, and two negative, *GSTP1* with *UGP2* and *NQO2* with UGT1A9.

The analysis of gene expression of phase II biotransformation showed two major processes dysregulated in CRC cells. The first is oxidative phosphorylation, a vital part of the cell’s metabolism and its main energy producer from FADH and NADH. NNMT takes part in nicotinamide biosynthesis in the cell, and NQO2 enables the reduction of two electrons in the electron transport chain. Hence, this aligns with the phase I expression pattern focused on oxidative stress, confirming the previous observation. The second process is glucuronic acid transfer, whose efficiency will be lowered in CRC cells as a consequence of the downregulation of the two described genes. Disruption of this process probably leads to the accumulation of first-phase products and inhibition of the detoxification process.

Differentially expressed transcripts in the third biotransformation phase (III) can be divided into two groups: adenosine triphosphate binding cassette (*ABCB2*, *ABCG2*, *ABCD3*, *ABCA8*) and solute carriers (*SLC2A3* and more than ten *SLC* highlighted). The expression of the *ABCB2* (*TAP1*) gene was upregulated in all four clinical stages, which confirms the analysis where *TAP1* exhibited a state of elevated expression in colon *adenocarcinoma*, too [73].

The next three mentioned adenosine triphosphate-binding cassette (*ABC*) transporter family members (*ABCG2*, *ABCD3*, *ABCA8*) had downregulated expression in the studied CRC samples in comparison to the control. The *ABCG2* gene encodes a protein that plays a protective role in capillary endothelial cells, the gastrointestinal tract, and hematopoietic stem cells, and it is expressed in many organs. Downregulated expression of *ABCG2* in normal and cancer tissues from colectomy specimens [74] was confirmed in CRC. It was stated that the decreased expression of this gene can be caused by the accumulation of genotoxins and the overproduction of nitric oxide [75]. *ABCD3* encodes an enzyme essential in the peroxisomal oxidation of lauric and palmitic acid. Large amounts of this gene were observed in the liver, kidneys, gut, brain microvessels, and skin [76,77]. Zhang et. al. also stated that the mRNA of *ABCD3* was decreased in CRC tissue in comparison to the control [78]. These results are in parallel with the CRC samples in the presented study. Hence, the expression of *ABCD3* may be considered as a potential diagnostic, prognostic, and therapeutic factor in CRC patients. The dramatically reduced expression of *ABCA8* in the CRC tissues is also confirmed by Yang et al. [79], where it was found that this gene inhibited CRC cell proliferation and metastasis through the Wnt/β-catenin signaling pathway, both in vitro and in vivo. Namely, downregulated expression of *ABCA8* induced epithelial transformation to mesenchyme via the ABCA8/ERK/ZEB1 pathway and promoted the progression of hepatocellular carcinoma [80]. All listed ATP-binding cassette mRNAs showed correlative activity (Table 7).

The second group of genes in phase III are solute carriers (*SLCs*), specifically *SLC2A3* encoding the glucose transporter (GLUT3) protein [81]. Upregulation of *SLC2A3* was stated in CRC. Overexpression of this gene was associated with progression and decreased survival in CRC [81,82]. A similar expression profile was noted for *SLC5A1* [83], *SLC6A14* [84], *SLC7A5* [85], *SLC12A2* [82], *SLC25A15* [32], *SLC25A32* [32,86], *SLCO1B3* [87], and *SLCO4A1* [88], which are enhanced in CRC, too. Furthermore, the results confirm the involvement of *SLC25A4* in the development of CRC and its silencing in all stages [32] and the reduced expression of *SLC35A1* in this tumor [87]. It was stated that mutations in *SLC25A15* and *SLC25A32* occurred at a higher frequency in patients with colon cancer than in healthy individuals [32]. Moreover, *SLC25A32* promotes tumor progression through MYC-mediated pathways [89]. However, no evidence for the involvement of other mRNAs was found. Regarding *AKRB7A2*, *SLC5A6*, and *SLC29A2*, for the first time, a significant role in CRC *adenocarcinoma* development as well as in a healthy colon is presented. The first was presented with silenced transcripts from phase I biotransformation. The next ones belong to phase III.

*SLC5A6* (SMVT) encodes a cotransporter sodium coupled to solutes as diverse as water-soluble vitamins, which can transport biotin and pantothenic acid. It was stated that *SLC5A6* may potentially be a diagnostic and prognostic biomarker in gastric cancer [90]. *SLC5A6* had increased expression in CRC. Correlative activity of this mRNA with others from phase III was observed in both healthy and cancerous colons. Apart from our results, there is no information on mRNA expression in CRC.

*SLC29A2* encodes human equilibrative nucleoside transporters (hENT2), which can transport nucleosides, nucleobases, and certain neurotransmitters across cell membranes. It was stated that SLC29A2 was expressed at lower levels in colon cancer cell lines originating from metastatic sites than from primary sites [91]. Expression was increased in the CSI stage and gradually reduced in the subsequent stages (CSII-IV) of CRC development, but it was still overexpressed. Correlative activity of this mRNA with others from phase III occurred in both healthy and cancerous colons.

The *AHR* (aryl hydrocarbon receptor)–ligand-dependent transcription factor regulates gene expression in lipid and cholesterol synthesis, xenobiotic (e.g., aryl hydrocarbons) metabolism, and many cellular responses not only in different physiological cells but also in all stages of cancer development (it can act as a tumor suppressor or tumor promoter) [92]. Its expression was found to be elevated in breast cancer (ER-negative), lung cancer (non-small cell), papillary thyroid carcinoma, cutaneous squamous cell carcinoma, T cell leukemia, B cell lymphoma, hepatocellular carcinoma, and glioblastoma, but in gastric cancer, it was downregulated [93]. The obtained results indicate that *adenocarcinoma* of CRC has a slightly elevated expression of the *AHR* in all clinical stages. Diminished *AHR* expression triggers cooperation with E2F1, which may lead to increased cell proliferation and apoptosis [94]. This gene had no correlations with other biotransformation-connected genes in healthy colon tissue (CC). The *AHR* in AC correlates negatively with *ADH1C* and with *AKR1B10* in CC; the higher the activity of *ADH1C* and/or *AKR1B10*, the lower the *AHR*. However, in CRC, its activity is negatively influenced by *DPEP1*, which is involved in leukotriene metabolism and inflammation. Furthermore, taking into account the obtained results of the analysis of AhR pathways (Table 2 and Table 4), it can be indicated that this transcript plays a pro-proliferative, apoptotic, and anti-inflammatory role in the development of CRC.

The presented data offer a novel insight into the transcriptional activity of genes related to the biotransformation process in phases I–III of CRC *adenocarcinoma* development, with additional insight into statistically significant transcripts of the canonical and non-canonical AhR pathways. An increase in the expression of genes related to the metabolism of lipids, retinol or other alcohols, hydroxy sterols, aldehydes, and carboxylic acids, in the absence of transcriptional activity of genes encoding coupling enzymes, may contribute to the induction of oxidative stress and “alleged inflammation” induced by the products of these reactions, such as reactive free radicals and non-metabolized compounds [95]. This is demonstrated by the differentiation in the gene expression profile shown in the study, not only in phases I and II but also in transporters (phase III). The profile of deregulated biotransformation genes disrupted processes, which are important for the intestinal tissue and are similar but also different from those in other tissues [96].

For the first time, the involvement of the phase I transcript *AKR7A2* and the phase III *SLC5A6* and *SLC29A2*, as well as those related to the AhR receptor pathways, was demonstrated in CRC development. Among the statistically significant genes associated with xenobiotic biotransformation, *AKR7A2* may metabolize acrolein and aflatoxins, and many other genes mentioned in the study are encoding enzymes involved in the biotransformation of compounds, such as leukotrienes, glutathione, lipids, retinol, glucose, vitamins B5 and B7, lipoate, and iodide; as well as transports of amino acids, nucleotides, and others, indicating that the development of CRC at the tumor site is associated with the disruption of the biotransformation process. The transcriptional activity of the 46 genes (phases I–III) related to the biotransformation/detoxification of xenobiotics/endobiotics and their connections to key pathways involved in carcinogenesis confirms the importance of this process in CRC development.

## 4. Materials and Methods

The project of examining samples of CRC was approved by the Bioethics Committee of the Medical University of Silesia with consent no. KNW/0022/KB1/21/I/10. All the procedures conform to the standards set by the Declaration of Helsinki (printed in the British Medical Journal in 1964 and the next revision). The participants provided understanding and written consent for the use of their resected material in the research project.

All participants, 98 patients aged 39–89 years, underwent surgical resection due to CRC at the Clinical Department of General, Colorectal, and Multiorgan Surgery of the Medical University of Silesia.

The material consisted of CRC tumors taken from resected large intestines and samples of untouched large intestines taken from the operating material, histologically checked as normal tissue. All the CRC *adenocarcinoma* sections were histopathologically and clinically described and divided into four clinical stages (CSI, CSII, CSIII, CSIV) according to the AJCC/UICC staging system, as shown in Table 8 [5,97,98].

Characteristics of the patients, basic parameters, and data collected from the interview are presented in Table 9.

The criteria for exclusion from the study group included patients who were diagnosed with a form of cancer other than CRC, had unclear histological confirmation of CRC, re-operated on due to the underlying disease, had genetic changes as well as metabolic or systemic conditions (such as obesity, hyperlipidemia, hyperglycemia), had previous radio- and/or chemotherapy, and those undergoing or had hormone replacement therapy completed within 5 years before the operation.

The criteria for inclusion were CRC *adenocarcinoma* in histopathological assessments of tumor material obtained intraoperatively from patients with different stages of disease undergoing elective classical surgical procedures and patients who lived all their lives in the industrial area of Silesia in Poland. Moreover, the inclusion criteria included patients who did not take any medications regularly. Forty cases met the criteria for inclusion.

### 4.1. Material for Analysis

**Figure 7:**
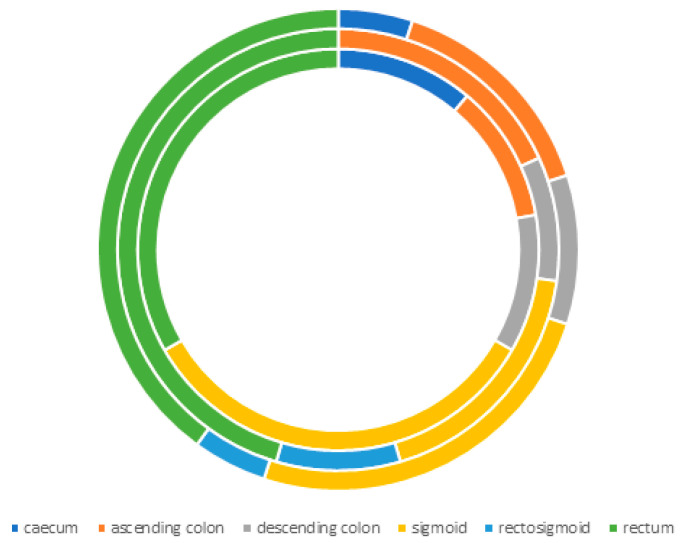
Distribution of tumor location in the colon (from the inside of the circle: female, male, and all).

The study material collected from the patients comprised a total of 98 biopsies from various parts of the colon. Of these, 20 biopsies from healthy colons and 20 biopsies from cancerous sites of the colon were selected for the study based on patient characteristics and inclusion and exclusion criteria. The percentage distribution of biopsies collected is presented in Figure 7.

The collected large intestine material consisted of cecums—5% (only female), ascending colons—5% (female) and 10% (male), descending colons—5% each, sigmoid—15% (female) and 10% (male), colons 5% (only male), and rectums—5% (female) and 25% (male), respectively.

After excision of the tumor and collection of samples for histopathological analysis, the material was preserved for molecular studies by storing it in a Dewar flask in containers in RNAprotect^®^ solution (QIAGEN GmbH, Hilden, Germany) at a low temperature of −80 °C, and then the samples were immediately transported to the molecular laboratory.

### 4.2. Histopathological Procedure

Tissues from all surgical biopsies were collected for histopathological examination. The tissues were fixed, dehydrated, embedded in paraffin, and thinly sliced using a microtome to prepare them for routine microscopic evaluation. Each tissue was stained with hematoxylin and eosin (H&E). The prepared slides were viewed under an Olympus AX70 microscope (Olympus Co., Tokyo, Japan). The slides were classified as *adenocarcinomas* of varying degrees of differentiation. The depth of invasion of the colon wall and surrounding tissues and the invasion of blood vessels or nerves were recorded, as indicated by the image. The microscopic images presented in this paper (Figure 1A–C and Figure S1–S3) were acquired using a NanoZoomer SQ slide scanner (Hamamatsu Photonic K.K., Hamamatsu, Shizuoka, Japan).

### 4.3. Material Grouping

**Figure 8:**
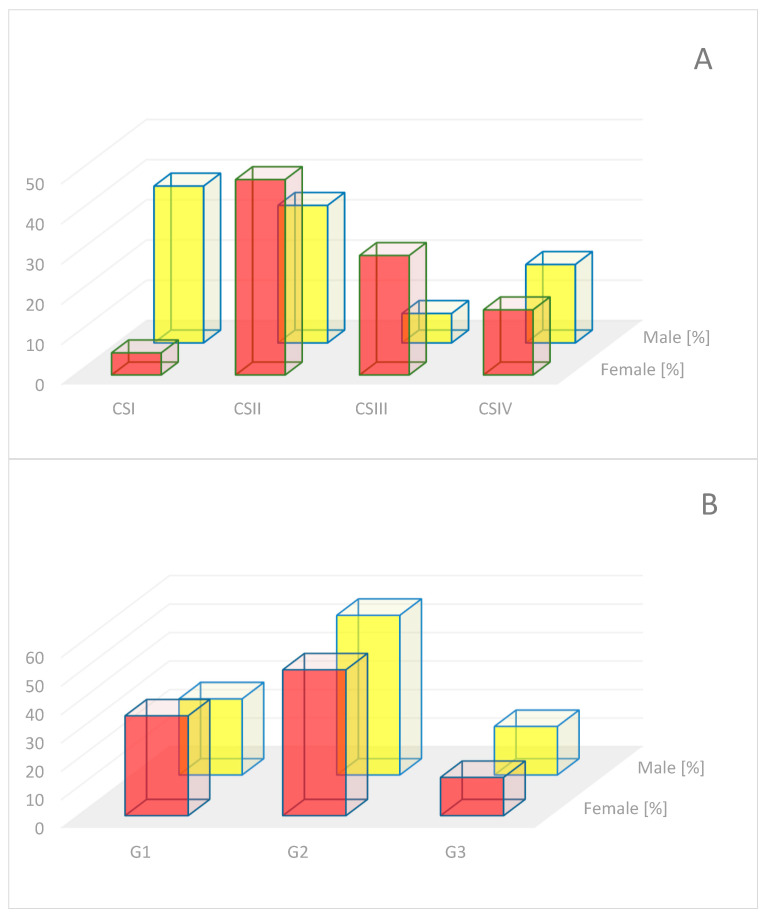
Distribution of clinical stages of colorectal cancer in patients (**A**) and histopathological grading of tumors (**B**).

The clinical stage of CRC (according to AJCC/UICC) was assessed intraoperatively during classical surgical techniques (without the use of electrical instruments) and with mechanical tissue compression kept to a minimum. Colorectal cancer specimens were obtained from 78 individuals (37 female and 41 male), and healthy colon tissues were obtained from 20 individuals (8 female and 12 male) hospitalized at the Clinical Department of General, Colorectal and Multiorgan Surgery in St Barbara Regional Specialist Hospital No 5 in Sosnowiec. The distribution of CRC clinical stages for females was as follows: CSI N = 2, CSII N = 18, CSIII N = 11, and CSIV N = 6. For males, it was CSI N = 16, CSII N = 14, CSIII N = 3, and CSIV N = 8. The percentage distribution of the clinical stages of colorectal cancer in patients and the distribution of histopathological differentiation grade of tumors (feature G) are presented in Figure 8A,B, respectively.

Based on the inclusion criteria of patients and taking into consideration the clinical stage, the tissues included in the experiment were divided into 5 groups: control (CC, N = 20), clinical stage I (CSI, N = 4; G1 and G2 (75%)), clinical stage II (CSII, N = 6; G2 (66%) and G1), clinical stage III (CSIII, N = 6; G2 (33%) and G3), and clinical stage IV (CSIV, N = 4; G3 and G2 (25%)), with the histopathological grade described.

### 4.4. RNA Extraction

The frozen postoperative control large colon (CC; histopathologically estimated) and CRC *adenocarcinoma* (histopathologically estimated) samples at different stages (CSI–CSIV) were homogenized separately with the use of TRIzol reagent and isolated total RNA in accordance with the producer’s protocol (Invitrogen Inc., Carlsbad, CA, USA). Isolated RNA was purified with RNeasy Mini Kit columns (QIAGEN, Germany). The purity of the isolated RNA was evaluated using standard methods recommended by the microarray manufacturer.

### 4.5. Microarray Analysis

The isolated RNA from each sample was used to prepare microarrays. Analysis was performed by the formation of cDNA and cRNA; next, its fragmentation and hybridization with HG-U133A microarrays were performed according to the producer’s manual (Affymetrix, Santa Clara, CA, USA). During all steps of analysis, the quality of cDNA and cRNA was checked according to the manufacturer’s recommendations. The fluorescence intensity signal was read using the Gene Chip Optical Scanner 3000 7G (Affymetrix, Santa Clara, CA, USA). The quality of the microarrays was checked in the Affymetrix^®^ GeneChip^®^ Command Console^®^ v6.1 Software (Figure S4). All data are deposited in the PL-Grid Infrastructure (www.plgrid.pl).

### 4.6. Selecting Gene Panels for Analysis

The examined selected panels of mRNA from HGU-133A microarrays of CRC (CSI –CSIV) and healthy colon tissue samples (CC) were based on separate genes associated with the biotransformation process (302 genes in the term “Cytochrome P450—arranged by substrate type” downloaded on 15 February 2020 and repeated in 2025), which correspond to 366 mRNAs on the used microarrays. Additionally, genes linked to the terms “I phase biotransformation”, “cytochrome P450”, “II phase biotransformation”, “III phase biotransformation”, “ATP binding cassette”, “transporters”, and “AhR canonical and non-canonical pathways” downloaded at the same time were investigated. The above-mentioned entries and the literature allowed us to create lists of probes (entities), which are presented in Tables S5–S10 and S17. Statistically significant entities were assigned their corresponding gene names and are presented in Tables S11–S16 and S18.

### 4.7. Statistical Analysis

The standard statistical analysis of the data obtained from microarrays was performed in the GeneSpring 13.0 software (Agilent Technologies, Inc., Santa Clara, CA, USA). Descriptive statistics were calculated for all mRNAs in each CRC clinical stage (CSI-IV) in comparison to the control colon tissues (CC). For comparison, all samples in all stages were subjected to analysis of variance with the *post hoc* HSD Tukey test with Benjamini–Hochberg correction (FDR—*False Discovery Rate*). The fold change (*FC*) parameter was calculated from statistically significant CRC mRNAs compared to the control.

The fluorescence signals of the mRNAs obtained from colorectal *adenocarcinoma* tissues (CRC in CSI–CSIV) and the control colon (CC) were normalized in MATLAB 8.0 software and submitted to the Excel application of Microsoft^®^Office. Statistical analysis of the Pearson correlations with a coefficient, r, from −1.00 (negative) to 1.00 (positive) of the significant genes in phases I–III was performed with the use of STATISTICA 13.3 (TIBCO) software. The significance level was set at *p* ≤ 0.05.

## 5. Conclusions

The presented disturbances in all studied phases (I–III) indicate that genes associated with biotransformation actively participate in the development of CRC. Potential diagnostic transcripts may include *ADH1C*, *GGT5*, *NQO2*, and *SLC25A5* in colorectal *adenocarcinoma*. Transcriptional activity of biotransformation genes in the healthy intestine was more intense in phases I–II and in phase III in CRC. The *AHR*, *EPHX1*, *GSTP1*, and *SLC25A32* were observed to be uncorrelated with other genes in the healthy intestine; they were only in CRC. Analysis of the transcriptional activity of genes associated with the biotransformation phases supports the theory that imbalances in subsequent phases of biotransformation may influence the development of CRC. The observed changes in the presented transcripts in phases I–III, as well as those associated with the AhR, indicate their importance for new therapies and directions for preventive measures in colorectal cancer.

The presented analysis demonstrates potential diagnostic and therapeutic targets for CRC limited to cellular biotransformation at the transcriptomic level. Further studies in vitro and in vivo are recommended.

## Figures and Tables

**Table 1:** Phases of the biotransformation process, panels of transcripts, and the number of significant mRNAs dependent on the *p*-value in the ANOVA test.

Phase	Panel	Number of mRNAs Dependent on *p*-Value			
		all	*p* < 0.05	*p* < 0.01	*p* < 0.001
I	Biotransformation	366	98	57	19
	Functionalization	121	29	19	6
	CYPs	91	9	1	0
II	Conjugation	75	14	11	7
III	ATP-binding cassette	69	11	4	1
	Transporters	456	63	22	11

Explanation: ANOVA—analysis of variance; ATP—adenosine triphosphate; CYP—cytochrome P450; *p*—probability.

**Table 2:** Significant mRNA of the biotransformation process in CSI—CIV of CRC.

Phase	Probe Set ID	Gene Symbol	FDR *p*-Value B-H Corrected	CSI vs. CC		CSII vs. CC		CSIII vs. CC		CSIV vs. CC	
				FC	Regulation	FC	Regulation	FC	Regulation	FC	Regulation
	200903_s_at	*AHCY*	3.5 × 10^−4^	1.55	up	2.77	up	1.08	up	1.61	up
I	206754_s_at	*CYP2B7P1*	0.003	1.09	up	2.26	up	2.19	up	4.45	up
	205983_at	*DPEP1*	0.004	3.03	up	2.30	up	2.34	up	3.69	up
	206262_at	***ADH1C***	0009	1.07	up	−4.73	down	−2.64	down	−4.78	down
	205582_s_at	***GGT5***	3.3 × 10^−4^	−1.16	down	1.35	up	2.01	up	2.05	up
	208847-s-at	*ADH5*	0.0002	−1.17	down	−1.43	down	−1.72	down	−2.44	down
	208848_at	*ADH5*	0.005	−1.95	down	−1.78	down	−1.57	down	−4.63	down
	206561_s_at	*AKR1B10*	0.03415	−1.27	down	−4.76	down	−4.01	down	−6.33	down
	202139__at	*AKR7A2*	0.005	−1.18	down	−1.39	down	−1.05	down	−2.42	down
	212224_at	*ALDH1A1*	1.50 × 10^−4^	−3.67	down	−7.04	down	−3.21	down	−6.74	down
	202017_at	*EPHX1*	0.005	−2.87	down	−1.39	down	−1.17	down	−2.05	down
	209368_at	*EPHX2*	0.0002	−1.68	down	−2.03	down	−1.84	down	−2.24	down
	212741_at	*MAOA*	3.61 × 10^−6^	−1.51	down	−3.38	down	−2.55	down	−4.78	down
	204389_at	*MAOA*	8.57 × 10^−8^	−1.61	down	−4.02	down	−2.61	down	−5.01	down
	204388_s_at	*MAOA*	1.65 × 10^−7^	−1.62	down	−3.27	down	−2.71	down	−4.19	down
	205127_at	*PTGS1*	0.007	−4.44	down	−3.14	down	−2.18	down	−4.43	down
	205128_x_at	*PTGS1*	0.008	−3.45	down	−2.55	down	−1.79	down	−3.10	down
	215813_s_at	*PTGS1*	0.006	−4.03	down	−2.99	down	−1.73	down	−4.30	down
II	200824_at	*GSTP1*	0.004	1.49	up	2.19	up	1.42	up	1.65	up
	202237_at	*NNMT*	4.6 × 10^−4^	1.95	up	2.8	up	5.5	up	4.51	up
	202238_s_at	*NNMT*	5.99 × 10^−4^	1.75	up	2.4	up	5.4	up	3.19	up
	203814_s_at	***NQO2***	0.004	−1.01	down	2.16	up	1.22	up	1.19	up
	204550_x_at	*GSTM1*	0.0008	−2.03	down	−1.77	down	−1.49	down	−1.34	down
	204418_at	*GSTM2*	0.0005	−2.31	down	−1.79	down	−1.44	down	−1.33	down
	204419_s_at	*GSTM4*	2.6 × 10^−5^	−1.95	down	−1.87	down	−2.48	down	−2.36	down
	203343_at	*UGDH*	0.013	−1.24	down	−2.14	down	−1.74	down	−2.68	down
	205480_s_at	*UGP2*	4.2 × 10^−6^	−1.96	down	−2.99	down	−2.37	down	−5.14	down
	221305_s_at	*UGT1A9*	0.013	−1.72	down	−2.68	down	−2.46	down	−1.55	down
	207245_at	*UGT2B17*	0.01184	−2.44	down	−8.52	down	−3.62	down	−5.93	down

Explanations: CRC—colorectal cancer; ID—identified number of transcript probes in the Affymetrix microarray; FDR—False Discovery Rate; B-H—Benjamini–Hochberg correction of *p*-value; CS—clinical stage of CRC tissues; CC—control tissue; FC—fold change.

**Table 3:** Significant mRNA of phase III in the CSI—CSIV stages of CRC.

Probe Set ID	Gene Symbol	FDR p-Value B-H Corrected	CSI vs. CC		CSII vs. CC		CSIII vs. CC		CSIV vs. CC	
			FC	Regulation	FC	Regulation	FC	Regulation	FC	Regulation
202307_s_at	*ABCB2*	0.0068	2.93	up	1.79	up	1.37	up	1.27	up
202497_x_at	*SLC2A3*	0.015	1.32	up	1.18	up	2.00	up	2.84	up
202498_s_at	*SLC2A3*	0.022	1.06	up	1.06	up	1.47	up	1.37	up
202499_s_at	*SLC2A3*	0.015	1.44	up	1.08	up	2.70	up	3.82	up
206628_at	*SLC5A1*	0.020	1.54	up	2.84	up	1.02	up	1.32	up
204087_s_at	*SLC5A6*	3.2 × 10^−4^	1.63	up	2.81	up	1.55	up	2.34	up
219795_at	*SLC6A14*	0.043	2.60	up	2.84	up	1.46	up	1.05	up
201195_s_at	*SLC7A5*	3.0 × 10^−5^	2.01	up	4.00	up	2.54	up	3.91	up
204404_at	*SLC12A2*	0.011	3.90	up	1.99	up	1.29	up	2.15	up
218653_at	*SLC25A15*	0.014	1.83	up	2.30	up	1.22	up	1.59	up
221020_s_at	*SLC25A32*	0.024	1.43	up	2.04	up	1.50	up	1.13	up
204717_s_at	*SLC29A2*	0.034	2.05	up	1.53	up	1.41	up	1.90	up
206354_at	*SLCO1B3*	0.026	1.40	up	1.86	up	1.07	up	2.41	up
219911_s_at	*SLCO4A1*	9.2 × 10^−6^	3.28	up	4.68	up	1.81	up	2.53	up
200657_at	***SLC25A5***	9.2 × 10^−4^	1.02	up	1.02	up	−1.65	down	−2.15	down
204719_at	*ABCA8*	4.6 × 10^−8^	−4.65	down	−7.15	down	−1.44	down	−8.08	down
202850_at	*ABCD3*	0.0315	−1.19	down	−1.36	down	−1.30	down	−2.99	down
209735_at	*ABCG2*	0.010	−3.20	down	−4.71	down	−3.78	down	−4.73	down
202825_at	*SLC25A4*	0.004	−2.00	down	−1.86	down	−1.81	down	−3.07	down
203306_s_at	*SLC35A1*	0.043	−1.17	down	−1.99	down	−1.49	down	−3.10	down

Explanations: CRC—colorectal cancer; ID—identified number of transcript probes in the Affymetrix microarray; FDR—False Discovery Rate; B-H—Benjamini–Hochberg correction of *p*-value; CS—clinical stage of CRC tissues; CC—control tissue; FC—fold change.

**Table 4:** Significant mRNA of AhR pathways in the CSI—CSIV stages of CRC.

Probe Set ID	Gene Symbol	FDR *p*-Value B-H Corrected	CSI vs. CC		CSII vs. CC		CSIII vs. CC		CSIV vs. CC	
			FC	Regulation	FC	Regulation	FC	Regulation	FC	Regulation
202820_at	*AHR*	0.014	1.21	up	1.15	up	1.51	up	1.30	up
206651_s_at	*CPB2*	7.4 × 10^−4^	1.11	up	1.06	up	1.09	up	1.30	up
2028_s_at	*E2F1*	0.032	1.03	up	1.24	up	1.05	up	1.27	up
204947_at	*E2F1*	0.001	1.40	up	1.09	up	1.04	up	1.54	up
203957_at	*E2F6*	2.0 × 10^−4^	1.29	up	1.61	up	1.05	up	1.13	up
206998_x_at	*PRB3*	0.0012	1.26	up	1.12	up	1.09	up	1.61	up

Explanations: CRC—colorectal cancer; ID—identified number of transcript probes in the Affymetrix microarray; FDR—False Discovery Rate; B-H—Benjamini–Hochberg correction of *p*-value; CS—clinical stage of CRC tissues; CC—control tissue; FC—fold change.

**Table 5:** Correlations between phase I transcripts of the biotransformation process.

CC vs. CC					CC vs. AC					AC vs. AC				
Gene	vs.	Gene	r	*p*	Gene	vs.	Gene	r	*p*	Gene	vs.	Gene	r	*p*
*ADH1C*	↔	*CYP2B7P1*	0.68	0.002	*AHCY*	↔	*ADH5*	−0.52	0.028	*DEPEP1*	↔	*AHR*	−0.49	0.040
*GGT5*	↔	*AHCY*	0.46	0.046	*DPEP1*	↔	*AHCY*	0.48	0.044	*GGT5*	↔	*ADH1C*	−0.61	0.007
*GGT5*	↔	*DPEP1*	0.50	0.030	*ADH1C*	↔	*AHR*	−0.56	0.016	*ADH5*	↔	*CYP2B7P1*	−0.47	0.047
*ADH5*	↔	*CYP2B7P1*	−0.58	0.010	*ADH5*	↔	*EPHX1*	0.48	0.045	*ADH5*	↔	*DEPEP1*	−0.59	0.010
*ADH5*	↔	*ADH1C*	−0.74	0.000	*AKRB10*	↔	*AHR*	−0.50	0.035	*AKRB10*	↔	*ADH1C*	0.77	0.000
*ADH5*	↔	*GGT5*	−0.50	0.029	*EPHX1*	↔	*GGT5*	0.54	0.022	*AKRB10*	↔	*GGT5*	−0.62	0.006
*AKRB10*	↔	*CYP2B7P1*	0.67	0.002	*EPHX1*	↔	*MAOA*	−0.69	0.002	*EPHX1*	↔	*GGT5*	0.56	0.016
*AKRB10*	↔	*ADH1C*	0.95	0.000	*MAOA*	↔	*MAOA*	0.54	0.022	*EPHX1*	↔	*AKR7A*	0.47	0.048
*AKRB10*	↔	*ADH5*	−0.82	0.000	*PTGS1*	↔	*MAOA*	−0.60	0.008	*EPHX2*	↔	*ADH1C*	0.58	0.011
*AKR7A2*	↔	*AHCY*	0.54	0.017						*MAOA*	↔	*ADH1C*	0.85	0.000
*AKR7A2*	↔	*CYP2B7P1*	0.53	0.020						*MAOA*	↔	*GGT5*	−0.70	0.001
*ALDH1A1*	↔	*CYP2B7P1*	−0.56	0.012						*MAOA*	↔	*ADH5*	0.50	0.035
*ALDH1A1*	↔	*ADH1C*	−0.72	0.001						*MAOA*	↔	*AKRB10*	0.71	0.001
*ALDH1A1*	↔	*GGT5*	−0.48	0.036						*PTGS1*	↔	*EPHX1*	0.52	0.027
*ALDH1A1*	↔	*ADH5*	0.92	0.000										
*ALDH1A1*	↔	*AKRB10*	−0.82	0.000										
*EPHX2*	↔	*CYP2B7P1*	0.66	0.002										
*EPHX2*	↔	*ADH1C*	0.63	0.004										
*EPHX2*	↔	*GGT5*	0.46	0.035										
*EPHX2*	↔	*ADH5*	−0.70	0.001										
*EPHX2*	↔	*AKRB10*	0.74	0.000										
*EPHX2*	↔	*AKR7A2*	0.52	0.023										
*EPHX2*	↔	*ALDH1A1*	−0.82	0.000										
*MAOA*	↔	*CYP2B7P1*	0.54	0.016										
*MAOA*	↔	*ADH1C*	0.54	0.018										
*MAOA*	↔	*AKRB10*	0.53	0.020										
*MAOA*	↔	*EPHX2*	0.50	0.031										
*PTGS1*	↔	*CYP2B7P1*	−0.58	0.010										
*PTGS1*	↔	*ADH1C*	−0.82	0.000										
*PTGS1*	↔	*ADH5*	0.76	0.000										
*PTGS1*	↔	*AKRB10*	−0.86	0.000										
*PTGS1*	↔	*ALDH1A1*	0.81	0.000										
*PTGS1*	↔	*EPHX2*	−0.75	0.000										
*PTGS1*	↔	*MAOA*	−0.76	0.000										

Explanations: CC, control colon; AC, *adenocarcinoma*; vs., versus; ↔ interdependence between individual mRNAs; −, negative correlation; r, correlation coefficient; *p*, probability < 0.05.

**Table 6:** Correlations between phase II transcripts of the biotransformation process.

CC vs. CC					CC vs. AC					AC vs. AC				
Gene	vs.	Gene	r	*p*	Gene	vs.	Gene	r	*p*	Gene	vs.	Gene	r	*p*
*NQO2*	↔	*GSTM1*	0.46	0.049	*GSTP1*	↔	*NQO2*	−0.55	0.017	*GSTP1*	↔	*UGP2*	−0.60	0.008
*NQO2*	↔	*GSTM2*	0.50	0.028	*NNMT*	↔	*UGT2B17*	0.48	0.046	*NQO2*	↔	*UGT1A9*	−0.53	0.023
*GSTM1*	↔	*GSTM2*	0.94	0.000	*NQO2*	↔	*NNMT*	0.51	0.032	*GSTM1*	↔	*GSTM2*	0.96	0.000
*NQO2*	↔	*GSTM4*	0.58	0.009	*GSTM2*	↔	*NNMT*	0.47	0.048	*UGDH*	↔	*UGP2*	0.54	0.021
*GSTM1*	↔	*UGDH*	−0.56	0.012	*GSTM4*	↔	*GSTM4*	0.58	0.013					
*GSTM2*	↔	*UGDH*	−0.67	0.002	*UGDH*	↔	*UGDH*	0.58	0.013					
*NNMT*	↔	*UGP2*	−0.62	0.005	*UGP2*	↔	*UGDH*	0.53	0.027					
*GSTM2*	↔	*UGP2*	−0.48	0.036	*UGT1A9*	↔	*NQO2*	−0.54	0.020					
*UGDH*	↔	*UGP2*	0.78	0.000	*UGT2B17*	↔	*UGT2B17*	0.52	0.027					
*GSTM1*	↔	*UGT1A9*	−0.57	0.011										
*GSTM2*	↔	*UGT1A9*	−0.66	0.002										
*UGDH*	↔	*UGT1A9*	0.78	0.000										
*GSTM1*	↔	*UGT2B17*	0.59	0.008										
*GSTM2*	↔	*UGT2B17*	−0.64	0.003										
*UGDH*	↔	*UGT2B17*	0.79	0.000										
*UGT1A9*	↔	*UGT2B17*	0.77	0.000										

Explanation: CC, control colon; AC, *adenocarcinoma*; vs.—versus; ↔ interdependence between individual mRNAs; − negative correlation; r, correlation coefficient; *p*, probability < 0.05.

**Table 7:** Significant correlations between phase III transcripts of the biotransformation process.

CC vs. CC					CC vs. AC					AC vs. AC				
Gene	vs.	Gene	r	p	Gene	vs.	Gene	r	p	Gene	vs.	Gene	r	p
*ABCB2*	↔	*SLC5A1*	0.88	0.000	*SLC5A1*	↔	*SLCO4A1*	0.52	0.028	*ABCB2*	↔	*SLCO4A1*	0.49	0.039
*ABCB2*	↔	*SLC5A6*	0.70	0.001	*SLC6A14*	↔	*SLC6A14*	0.55	0.018	*SLC2A3*	↔	*SLC5A1*	−0.56	0.016
*ABCB2*	↔	*SLC12A2*	0.65	0.004	*SLC6A14*	↔	*SLC25A15*	0.49	0.037	*SLC2A3*	↔	*SLC25A5*	−0.82	0.000
*ABCB2*	↔	*SLC25A5*	0.72	0.001	*SLC7A5*	↔	*SLC2A3*	0.47	0.048	*SLC2A3*	↔	*SLCO4A1*	−0.52	0.028
*ABCB2*	↔	*SLC25A15*	0.78	0.000	*SLC7A5*	↔	*SLC5A1*	−0.53	0.023	*SLC2A3*	↔	*ABCA8*	−0.61	0.008
*ABCB2*	↔	*SLC29A2*	0.77	0.000	*SLC7A5*	↔	*ABCA8*	−0.55	0.019	*SLC5A1*	↔	*SLC6A14*	0.61	0.008
*ABCB2*	↔	*SLCO4A1*	0.37	0.014	*SLC12A2*	↔	*ABCD3*	0.52	0.026	*SLC5A1*	↔	*SLC25A5*	0.55	0.017
*ABCB2*	↔	*ABCD3*	0.60	0.009	*SLC25A5*	↔	*SLCO1B3*	−0.63	0.005	*SLC5A1*	↔	*SLC25A15*	0.66	0.003
*ABCB2*	↔	*ABCG2*	−0.73	0.001	*SLC25A15*	↔	*ABCD3*	0.55	0.019	*SLC5A1*	↔	*SLC25A32*	0.48	0.045
*ABCB2*	↔	*SLC25A4*	−0.73	0.001	*SLC29A2*	↔	*SLCO4A1*	0.47	0.049	*SLC5A1*	↔	*SLCO4A1*	0.66	0.003
*SLC2A3*	↔	*SLC7A5*	0.57	0.013	*SLC29A2*	↔	*ABCD3*	0.50	0.036	*SLC5A1*	↔	*ABCA8*	0.47	0.048
*SLC2A3*	↔	*SLC25A5*	0.47	0.050	*SLCO1B3*	↔	*SLCO4A1*	0.57	0.014	*SLC5A6*	↔	*SLC7A5*	0.73	0.001
*SLC2A3*	↔	*ABCD3*	−0.49	0.040	*SLC25A5*	↔	*SLC35A1*	0.62	0.006	*SLC5A6*	↔	*SLC29A2*	0.65	0.004
*SLC5A1*	↔	*SLC5A6*	0.78	0.000	*ABCA8*	↔	*SLC2A3*	−0.48	0.043	*SLC5A6*	↔	*SLC25A5*	−0.78	0.000
*SLC5A1*	↔	*SLC12A2*	0.73	0.001	*ABCD3*	↔	*SLCO4A1*	0.52	0.026	*SLC6A14*	↔	*SLC25A32*	0.49	0.037
*SLC5A1*	↔	*SLC25A5*	0.79	0.000	*ABCG2*	↔	*SLCO4A1*	−0.59	0.010	*SLC6A14*	↔	*ABCA8*	0.57	0.013
*SLC5A1*	↔	*SLC25A15*	0.87	0.000	*SLC25A4*	↔	*SLCO4A1*	−0.59	0.010	*SLC6A14*	↔	*ABCD3*	0.48	0.045
*SLC5A1*	↔	*SLC29A2*	0.83	0.000	*SLC35A1*	↔	*ABCD3*	0.51	0.031	*SLC7A5*	↔	*SLC29A2*	0.61	0.007
*SLC5A1*	↔	*SLCO4A1*	0.56	0.015						*SLC7A5*	↔	*SLCO4A1*	0.47	0.048
*SLC5A1*	↔	*SLC25A5*	−0.58	0.012						*SLC7A5*	↔	*SLC25A5*	−0.66	0.003
*SLC5A1*	↔	*ABCD3*	0.65	0.003						*SLC7A5*	↔	*ABCG2*	−0.58	0.011
*SLC5A1*	↔	*ABCG2*	−0.82	0.000						*SLC7A5*	↔	*SLC25A4*	−0.58	0.011
*SLC5A1*	↔	*SLC25A4*	−0.82	0.000						*SLC12A2*	↔	*SLC25A15*	0.54	0.020
*SLC5A6*	↔	*SLC12A2*	0.59	0.010						*SLC12A2*	↔	*SLC29A2*	0.58	0.012
*SLC5A6*	↔	*SLC25A5*	0.58	0.013						*SLC25A5*	↔	*SLCO4A1*	0.50	0.033
*SLC5A6*	↔	*SLC25A15*	0.79	0.000						*SLC25A5*	↔	*ABCA8*	0.63	0.005
*SLC5A6*	↔	*SLC29A2*	0.77	0.000						*SLC25A15*	↔	*SLC25A32*	0.56	0.017
*SLC5A6*	↔	*SLCO4A1*	0.61	0.007						*SLC29A2*	↔	*SLC25A5*	−0.67	0.002
*SLC5A6*	↔	*ABCD3*	0.47	0.047						*SLC29A2*	↔	*ABCG2*	−0.51	0.030
*SLC5A6*	↔	*ABCG2*	−0.66	0.003						*SLC29A2*	↔	*SLC25A4*	−0.51	0.030
*SLC5A6*	↔	*SLC25A4*	−0.66	0.003						*SLCO4A1*	↔	*SLC25A5*	−0.60	0.009
*SLC6A14*	↔	*SLCO1B3*	0.62	0.006						*SLC25A5*	↔	*ABCG2*	0.55	0.018
*SLC7A5*	↔	*SLCO4A1*	0.67	0.003						*SLC25A5*	↔	*SLC25A4*	0.55	0.018
*SLC7A5*	↔	*ABCA8*	0.69	0.002						*ABCA8*	↔	*ABCD3*	0.60	0.008
*SLC7A5*	↔	*SLC35A1*	−0.70	0.001						*ABCA8*	↔	*ABCG2*	0.52	0.027
*SLC12A2*	↔	*SLC25A5*	0.66	0.003						*ABCA8*	↔	*SLC25A4*	0.52	0.027
*SLC12A2*	↔	*SLC25A15*	0.83	0.000						*ABCA8*	↔	*SLC35A1*	0.78	0.000
*SLC12A2*	↔	*SLC29A2*	0.74	0.000						*ABCD3*	↔	*SLC35A1*	0.51	0.032
*SLC12A2*	↔	*ABCA8*	0.64	0.004						*ABCG2*	↔	*SLC35A1*	0.56	0.016
*SLC12A2*	↔	*ABCD3*	0.61	0.007						*SLC35A1*	↔	*SLC35A1*	0.56	0.016
*SLC12A2*	↔	*ABCG2*	−0.81	0.000										
*SLC12A2*	↔	*SLC25A4*	−0.81	0.000										
*SLC12A2*	↔	*SLC35A1*	0.73	0.001										
*SLC25A5*	↔	*SLC25A15*	0.73	0.001										
*SLC25A5*	↔	*SLC29A2*	0.72	0.001										
*SLC25A5*	↔	*SLC25A5*	−0.53	0.024										
*SLC25A5*	↔	*ABCA8*	0.47	0.048										
*SLC25A5*	↔	*ABCD3*	0.73	0.001										
*SLC25A5*	↔	*ABCG2*	−0.68	0.002										
*SLC25A5*	↔	*SLC25A4*	−0.68	0.002										
*SLC25A15*	↔	*SLC29A2*	0.86	0.000										
*SLC25A15*	↔	*SLCO4A1*	0.52	0.026										
*SLC25A15*	↔	*ABCA8*	0.48	0.044										
*SLC25A15*	↔	*ABCD3*	0.59	0.010										
*SLC25A15*	↔	*ABCG2*	−0.77	0.000										
*SLC25A15*	↔	*SLC25A4*	−0.77	0.000										
*SLC25A15*	↔	*SLC35A1*	0.54	0.021										
*SLC29A2*	↔	*ABCA8*	0.61	0.008										
*SLC29A2*	↔	*ABCD3*	0.80	0.000										
*SLC29A2*	↔	*ABCG2*	−0.90	0.000										
*SLC29A2*	↔	*SLC25A4*	−0.90	0.000										
*SLC29A2*	↔	*SLC35A1*	0.63	0.005										
*ABCA8*	↔	*ABCD3*	0.78	0.000										
*ABCA8*	↔	*ABCG2*	−0.66	0.003										
*ABCA8*	↔	*SLC25A4*	−0.66	0.003										
*ABCA8*	↔	*SLC35A1*	0.90	0.000										
*ABCD3*	↔	*ABCG2*	−0.81	0.000										
*ABCD3*	↔	*SLC25A4*	−0.81	0.000										
*ABCD3*	↔	*SLC35A1*	0.68	0.002										
*ABCG2*	↔	*SLC25A4*	1.00	0.000										
*ABCG2*	↔	*SLC35A1*	−0.63	0.005										
*SLC25A4*	↔	*SLC35A1*	−0.63	0.005										

Explanation: CC, control colon; AC, *adenocarcinoma*; vs.—versus; ↔ interdependence between individual mRNAs; − negative correlation; r, correlation coefficient; *p*, probability < 0.05.

**Table 8:** Descriptions of clinical stages.

Clinical Stage	Description
CSI	The cancer cells invade the submucosal muscular layer of the colon. They have not spread into nearby tissues or lymph nodes (T1 or T2, N0, M0).
CSII	The cancer has grown through the layers of the muscle to the lining of the abdomen and has grown into the visceral peritoneum. It has not spread to the nearby lymph nodes or elsewhere (T4, N0, M0).
CSIII	The cancer has grown through the bowel wall or to surrounding organs and into lymph nodes or to a nodule of tumor in tissues around the colon or rectum that do not appear to be lymph nodes. The cancer has grown through the bowel wall or to the pericolorectal tissue or directly to the surrounding organs and into lymph nodes. The cancer has not spread to distant organs (T1 or T2, N1 or N1c, M0; or T1, N2a, M0T3 or T4a, N1 or N1c, M0; T2 or T3, N2a, M0; or T1 or T2, N2b, M0; T4a, N2a, M0; T3 or T4a, N2b, M0; or T4b, N1 or N2, M0).
CSIV	The cancer has spread to a distant part of the body, such as the liver and more than 1 part of the body or to the peritoneum (any T, any N, M1x a b c).

Legend: T—tumor, N—node, M—metastasis.

**Table 9:** Characteristics of patients included in the test.

Parameters	Indication or Range
Age	39–89 years
Male {%]	55%
BMI [kg/m^2^]	22.1–24.6
Systolic blood pressure [mmHg]	115–145
Diastolic blood pressure [mmHg]	80–90
Hemoglobin [g/dL]	8–11
WBC × 10^3^/µL	5–10.8
Cholesterol [mg/dL]	180–240
Fasting glycemia [mg/dL]	75–120
CRP [mg/dL]	1–6
Regular exercise	None
Socioeconomic status	Good
Health	Early stage (CSI and CSII) of CRC—flatulency, gastrointestinal pricking; late stage (CSIII and CSIV) of CRC—general weakness
Alcohol consumption	Occasionally
Smoking	Smoking stopped at least 5–15 years earlier
Diet	Often meat
Eating grilled food	Occasionally
Sedentary lifestyle	Often, all work and watch TV
Exposure to other carcinogens	Environmental, connected to living from birth in highly industrialized areas (steel mills, mines, cars; exceeding air pollution standards)

Legend: BMI—Body Mass Index, WBC—White Blood Cell, CRP—C-Reactive Protein.

## Data Availability

All data are deposited in the PL-Grid Infrastructure (www.plgrid.pl).

## Supplementary Materials

The following supporting information can be downloaded at https://www.mdpi.com/article/10.3390/ijms262412116/s1.

## Abbreviations

The following abbreviations are used in this manuscript:

ABC	Adenosine triphosphate-binding cassette
AC	Adenocarcinoma
ADH	Alcohol dehydrogenase
ALDH	Aldehyde dehydrogenase
ANOVA	Analysis of variance
BaP	Benzo(a)pyrene
B-H	Benjamini–Hochberg correction of *p*-value
BRAF	B-rapidly accelerated fibrosarcoma
CA 19-9	Carbohydrate antigen 19-9
CAR T	Chimeric antigen receptor T cells
CC	Control colon
CEA	Carcinoembryonic antigen
CRC	Colorectal cancer
CS	Clinical stage
CYP	Cytochrome P450
down	downregulation
EGFR	Epidermal growth factor receptor
ERBB2	Erythroblastic oncogene B
FC	Fold Change
FDR	False discovery rate
FMO	Flavin-containing monooxidase
G1	Grade 1
G2	Grade 2
G3	Grade 3
GST	Glutathione S-transferase
HER2	Human epidermal growth factor receptor 2
HSD	High Significant Difference
ID	Identified number of transcript probes in Affymetrix microarrays
KRAS	Kirsten Rat sarcoma
MAOA	Monoamine oxidase
MEK	Mitogen-activated protein kinase kinase
NRAS	Neuroblastoma RAS
*p*-value	Probability value
r	Correlation coefficient
RAS	Rat Sarcoma
S	Supplemental
SLC	Solute Carrier transporter
TCDD	2,3,7,8-tetrachlorodibenzo-p-dioxin
TNM	Tumor Node Metastasis
UGT	Uridine diphospho-glucuronosyl ransferase
up	upregulation
VEGF	Vascular endothelial growth factor
	Names of genes used in the text are explained in Table S18.
