# Supplementary material for: Transcriptional Activity of Genes Related to the Biotransformation Process in the Development of Colorectal Cancer
Source: Int J Mol Sci. 2025 Dec 16;26(24):12116. doi: 10.3390/ijms262412116 (PMC12733395; doi:10.3390/ijms262412116)
Supplement: Supplementary file 1 [file ijms-26-12116-s001.zip › Supplementary material/S18 Significant genes name and functions.pdf]

Table S18. Differentially expressed genes in the biotransformation process in the CRC tissues, their name and functions (https://www.ncbi.nlm.nih.gov/gene)

Phase	Gene	Name	Function
I	*AHCY*	S-adenosylhomocysteine hydrolase	Gene encodes an enzyme which converts S-adenosylhomocysteine (SAH) into adenosine and L-homocysteine; regulates the addition of methyl groups to other compounds (methylation process).
	CYP2B7P1	Cytochrome P450, Family 2, Subfamily B, Polypeptide 7 Pseudogene 1	Gene encodes an enzyme that plays a crucial role in metabolizing various xenobiotics, among others, nicotine. CYP2B mRNA was detected in many human cells, also in the intestine.
	DPEP1	Dipeptidase 1	Gene encodes an enzyme that acts as a zinc-dependent metalloproteinase, hydrolyzing various dipeptides, and it plays a key role in converting leukotriene; as an adhesion molecule can recruit neutrophils to inflamed tissues; in cancer can act as either an oncogene promoter or a tumor suppressor.
	ADH1C	Alcohol dehydrogenase 1C	Gene encodes an enzyme which metabolizes alcohol to acetaldehyde; also metabolizes other aliphatic alcohols, retinol, hydroxysteroids, and lipid peroxidation products.
	GGT5	Gamma-glutamyltransferase 5	Gene encodes an enzyme which hydrolyzes the gamma-glutamyl moiety of glutathione; impacts cellular redox states, detoxification, and inflammatory responses
	ADH5	Alcohol dehydrogenase 5 (class III)	Gene encodes an enzyme which metabolizes ethanol, retinol, other aliphatic alcohols, hydroxysteroids, and lipid peroxidation products.
	AKR1B10	Aldo-keto reductase family 1 member B10	Gene encodes an enzyme which catalyzes the reduction of aldehydes and ketones, many carbonyls; it takes part in the synthesis of lipids.
	AKR7A2	Aldo-keto reductase family 7 member A2	Gene encodes an enzyme that detoxifies aldehydes, ketones, aflatoxin B1, and 2-carboxybenzaldehyde.
	ALDH1A1	Aldehyde Dehydrogenase 1 Family Member A1	Gene encodes an enzyme which oxidizes retinal (retinaldehyde) to retinoic acid; it catalyzes the oxidation of acetaldehyde to acetic acid; high activity is a key marker for cancer stem cells.
	EPHX1	Epoxide hydrolase 1	Gene encodes an enzyme which converts epoxides into less reactive dihydrodiols; it detoxifies harmful substances, such as those found in tobacco smoke and pollution; metabolizes endogenous lipids, which can influence processes like inflammation and blood vessel function.

	EPHX2	Epoxide hydrolase 2	Gene encodes an enzyme that hydrolyzes epoxides into less active diols and has lipid phosphate phosphatase activity. Its functions include regulating blood vessel pressure, managing inflammatory responses, and playing a role in the breakdown of lipids and cholesterol.
	MAOA	Monoamine oxidase	Gene encodes an enzyme that affects the activity of serotonin, dopamine, and norepinephrine.
	PTGS1	Prostaglandin-Endoperoxide Synthase 1	Gene encodes an enzyme that produces prostaglandins, which are important in the clotting process and regulation of blood pressure.
II	*GSTP1*	Glutathione S-transferase pi 1	Gene encodes an enzyme which detoxifies a wide variety of electrophilic compounds by catalyzing their conjugation with glutathione, and plays roles in signal transduction pathways such as the c-Jun N-terminal kinase (JNK) cascade.
	NNMT	Nicotinamide N-methyltransferase	Gene encodes an enzyme which catalyzes the methylation of nicotinamide, using S-adenosyl-L-methionine, resulting in the production of 1-methylnicotinamide.
	NQO2	NAD(P)H dehydrogenase [quinone] 2	Gene encodes an enzyme that catalyzes the two-electron reduction of quinones, making them more soluble and easier to excrete from the body.
	GSTM1	Glutathione-S-transferase mu 1	Gene encodes an enzyme which detoxifies harmful substances (carcinogens and products of oxidative stress), conjugating them with glutathione.
	GSTM2	Glutathione-S-transferase mu 2	Gene encodes an enzyme that detoxifies carcinogens and other harmful substances, helping protect cells from damage (conjugates them to glutathione)s.
	GSTM4	Glutathione-S-transferase mu 4	Gene encodes an enzyme that detoxifies endogenous and exogenous electrophilic compounds through conjugation with glutathione.
	UGDH	UDP-glucose 6-dehydrogenase	Gene encodes an enzyme which converts UDP-glucose, a critical component for the synthesis of glucosaminoglycans, hyaluronic acid, and other components of the extracellular matrix.
	UGP2	UDP-glucose pyrophosphorylase 2	Gene encodes an enzyme which transfers a glucose moiety from glucose-1-phosphate to MgUTP and forms UDP-glucose and MgPPi. In liver and muscle tissue, UDP-glucose is a direct precursor of glycogen.
	UGT1A9	UDP glucuronosyltransferase 1A9	Gene encodes an enzyme that converts small, lipophilic molecules like drugs, hormones, and toxins into water-soluble metabolites that can be easily excreted from the body.
	UGT2B17	UDP-glucuronosyltransferase 2B17	Gene encodes an enzyme that glucuronidates steroid hormones, drugs, and other lipid-soluble compounds.

III	*ABCB2*	ATP-binding cassette transporter B2	Gene encodes a protein that is a part of transporters that transport fragments of bacterial or viral peptides (generated from protein degradation) from the cytoplasm into the endoplasmic reticulum.
	ABCD3	ATP-binding cassette transporter D3	Gene encodes a peroxisomal membrane transporter protein, which plays a crucial role in lipid metabolism
	ABCA8	ATP-binding cassette transporter A8	Gene encodes a transporter protein, which plays a role in cholesterol efflux and other lipids.
	ABCG2	ATP-binding cassette transporter G2	Gene encodes a transporter protein, which excretes uric acid in the urine and transports drugs out of cells. It plays a role in protecting cells from toxins and potentially influencing how the body responds to certain medications.
	SLC2A3	Solute carrier family 2 member 3	Gene encodes a transporter protein, which is implicated in glucose transport from outside the cell to the inside, down a concentration gradient.
	SLC5A1	Solute carrier family 5 member 1	Gene encodes the SGLT1 sodium-dependent glucose transporter that absorbs glucose and galactose from the diet. It works by coupling the transport of these sugars into intestinal cells with the movement of sodium ions, also helping to transport water.
	SLC5A6	Solute carrier family 5 member 6	Gene encodes the sodium-dependent multivitamin transporter (SMVT), a protein responsible for transporting biotin, pantothenic acid, and lipoate into cells and across the blood-brain barrier.
	SLC6A14	Solute carrier family 6 member 14	Gene encodes a protein transporter called ATB0,+, that imports amino acids into cells. Its primary functions include nutrient absorption in the gut, maintaining amino acid balance in tissues like the lungs, and playing a role in various diseases.
	SLC7A5	Solute carrier family 7 member 5	Gene encodes a protein known as LAT1 that transports a sodium-independent amino acid. It exchanges large neutral amino acids, such as leucine and phenylalanine, for intracellular glutamine.
	SLC12A2	Solute carrier family 12 member 2	Gene encodes a protein that transports sodium, potassium, and chloride ions across cell membranes (mediates sodium and chloride transport and reabsorption*)*. This protein, also known as NKCC1 (Na-K-2Cl cotransporter 1), is essential for regulating cell volume and establishing the proper concentration of ions inside cells.
	SLC25A15	Solute carrier family 25 member 15	Gene encodes the mitochondrial ornithine transporter 1 protein, which is crucial for the urea cycle, because it transports ornithine from the cytosol into the mitochondrial matrix, a necessary step to remove toxic ammonia from the body.

	SLC25A32	Solute carrier family 25 member 32	Gene encodes a protein transporter that transports tetrahydrofolate (THF) as well as FAD into mitochondria and regulates mitochondrial one-carbon metabolism and redox balance.
	SLC29A2	Solute carrier family 29 member 2	Gene encodes a protein transporter that transports nucleosides for nucleotide synthesis by salvage pathways in cells that lack *de novo* biosynthetic pathways. Nucleoside transport plays a key role in the regulation of many physiologic processes through its effect on adenosine concentration at the cell surface.
	SLCO1B3	Solute carrier organic anion family 1 member B3	Gene encodes a protein, the OATP1B3 transmembrane transporter that mediates the sodium-independent uptake of endogenous and xenobiotic compounds and plays a critical role in bile acid and bilirubin transport from the blood into liver cells for detoxification and excretion.
	SLCO4A1	Solute carrier organic anion transporter family member 4A1	Gene encodes a protein, the OATP4A1 transmembrane transporter that moves thyroid hormones, steroid conjugates, estrogens, bile acids, and certain drugs into cells (sodium-independent), across membranes in organs such as the kidney, liver, and placenta.
	SLC25A5	Solute carrier family 25 member 5	Gene encodes a mitochondrial protein called ADP/ATP translocase2 (ANT2), which is a key component of the inner mitochondrial membrane. It exchange of ADP (from the cytoplasm) and ATP (from the mitochondria), linking cellular energy production and utilization.
	SLC25A4	Solute carrier family 25 member 4	Gene encodes the adenine nucleotide translocator 1 (ANT1), which transports ADP into the mitochondrial matrix and ATP out into the cytoplasm (energy production and energy homeostasis).
	SLC35A1	Solute carrier family 35 member 1	Gene encodes a protein of the Golgi apparatus membrane, which transports cytidine monophosphate-sialic acid (CMP-sialic acid) from the cytosol into the Golgi. This process is crucial for glycosylation, the process of adding sugars to proteins and lipids, which is essential for cell signaling, cell-cell interactions, and immune responses.
AHR paths	*AHR*	Aryl Hydrocarbon Receptor	Gene encodes a transcription factor that regulates gene expression in response to external and internal signals. The products/enzymes involved in the detoxification of environmental chemicals.
	CPB2	Carboxypeptidase B2 (CPU, PCPB, TAFI)	Gene encodes an enzyme, carboxypeptidase B2, which hydrolyzes C-terminal peptide bonds and cleaves basic amino residues (which are important for the binding and activation of plasminogen).

	E2F1	E2F transcription factor 1	Gene encodes a transcription factor that regulates cell cycle, DNA repair, and apoptosis. It activates genes necessary for cell growth and DNA replication, promotes DNA damage repair, and influences apoptosis
	E2F6	E2F transcription factor 6	Gene encodes a protein that inhibits E2F-dependent transcription, playing a role in cell cycle regulation.
	PRB3	Proline-rich protein BstNI subfamily 3	Gene encodes a salivary glycoprotein that is recognized as the "first line of oral defense" against the detrimental effects of polyphenols in the diet and pathogen infections.
