# Supplementary material for: Transcriptional Activity of Genes Related to the Biotransformation Process in the Development of Colorectal Cancer
Source: Int J Mol Sci. 2025 Dec 16;26(24):12116. doi: 10.3390/ijms262412116 (PMC12733395; doi:10.3390/ijms262412116)
Supplement: Supplementary file 1 [file ijms-26-12116-s001.zip › Supplementary material/Supplementary materials check list.pdf]

Supplementary materials for „Transcriptional Activity of Genes Related to the Biotransformation Process in the Development of Colorectal Cancer."

S1 Histopathological image of CRC in G1 (10x) with dirty necrosis

S2 Histopatological image of CRC in G2 (10x) primitive light, desmoplasm

S3 Histopatological image of CRC in G3 (20x) primitive colon cancer cells

S4 Figure of quality of microarrays

S5 List of 366 entities of biotransformation

S6 List of 121 entities of functionalization

S7 List of 91 entities of cytochrome P450 (CYPs)

S8 List of 75 entities of conjugation

S9 List of 69 entities of ATP Binding Cassette

S10 List of 456 entities of transporters

S17 List of 73 entities of AHR canonical and non-canonical pathways

S11 ANOVA results of 98 significant from 366 entities of biotransformation

S12 ANOVA results of 29 significant mRNAs from 121 entities of functionalization

S13 ANOVA results of 9 significant mRNAs from 91 CYPs

S14 ANOVA results of 14 significant mRNAs from 75 conjugation

S15 ANOVA results of 11 significant mRNA from 69 ATP-binding cassette

S16 ANOVA results of 63 significant mRNA from 456 transporters

S18 ANOVA results of 17 significant mRNA of AHR path ANOVA

S19 Significant gene names and functions

S20 Correlations of phase I transcripts distinguished in the normal control large colon tissue (CC)

S21 Correlations of phase I transcripts distinguished in normal control large colon tissue (CC) vs in adenocarcinoma tissue (AC)

S22 Correlations of phase I transcripts distinguished in adenocarcinoma tissue (AC vs AC)

S23 Correlations of phase II transcripts distinguished in normal control large colon tissue (CC vs CC)

S24 Correlations of phase II transcripts distinguished in normal control large colon tissue (CC) vs in adenocarcinoma tissue (AC)

S25 Correlations of phase II transcripts distinguished in adenocarcinoma tissue (AC vs AC)

S26 Correlations of phase III transcripts distinguished in the normal control large colon tissue (CC)

S27 Correlations of phase III transcripts distinguished in the normal control large colon tissue (CC) vs in adenocarcinoma tissue (AC)

S28 Correlations of phase III transcripts distinguished in adenocarcinoma tissue (AC vs AC)

S29 Correlations of phase I and II transcripts distinguished in the normal control large colon tissue (CC)

S30 Correlations of phase II and III transcripts distinguished in normal control large colon tissue (CC)

S31 Correlations of phase I and III transcripts distinguished in the normal control large colon tissue (CC)

S32 Correlations of phase I and II transcripts distinguished in adenocarcinoma tissue (AC vs AC)

S33 Correlations of phase II and III transcripts distinguished in adenocarcinoma tissue (AC vs AC)

S34 Correlations of phase I and III transcripts distinguished in adenocarcinoma tissue (AC vs AC)
