# Supplementary figures and images for: Transcriptional Activity of Genes Related to the Biotransformation Process in the Development of Colorectal Cancer
Source: Int J Mol Sci. 2025 Dec 16;26(24):12116. doi: 10.3390/ijms262412116 (PMC12733395; doi:10.3390/ijms262412116)

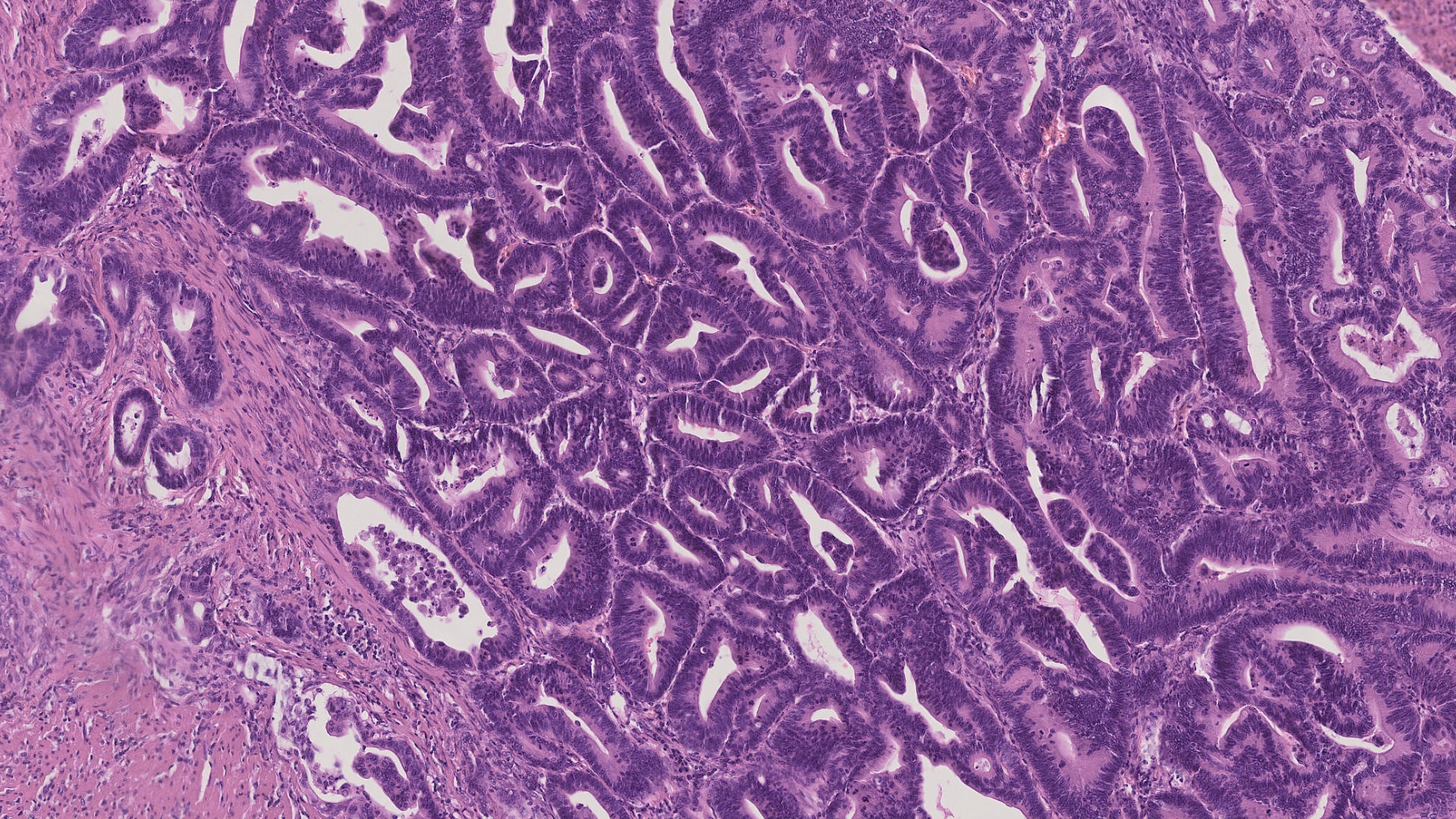

Supplement: Supplementary file 1 [file ijms-26-12116-s001.zip › Supplementary material/S1 Histopathological image of CRC in G1 (10x) with dirty necrosis.jpg]

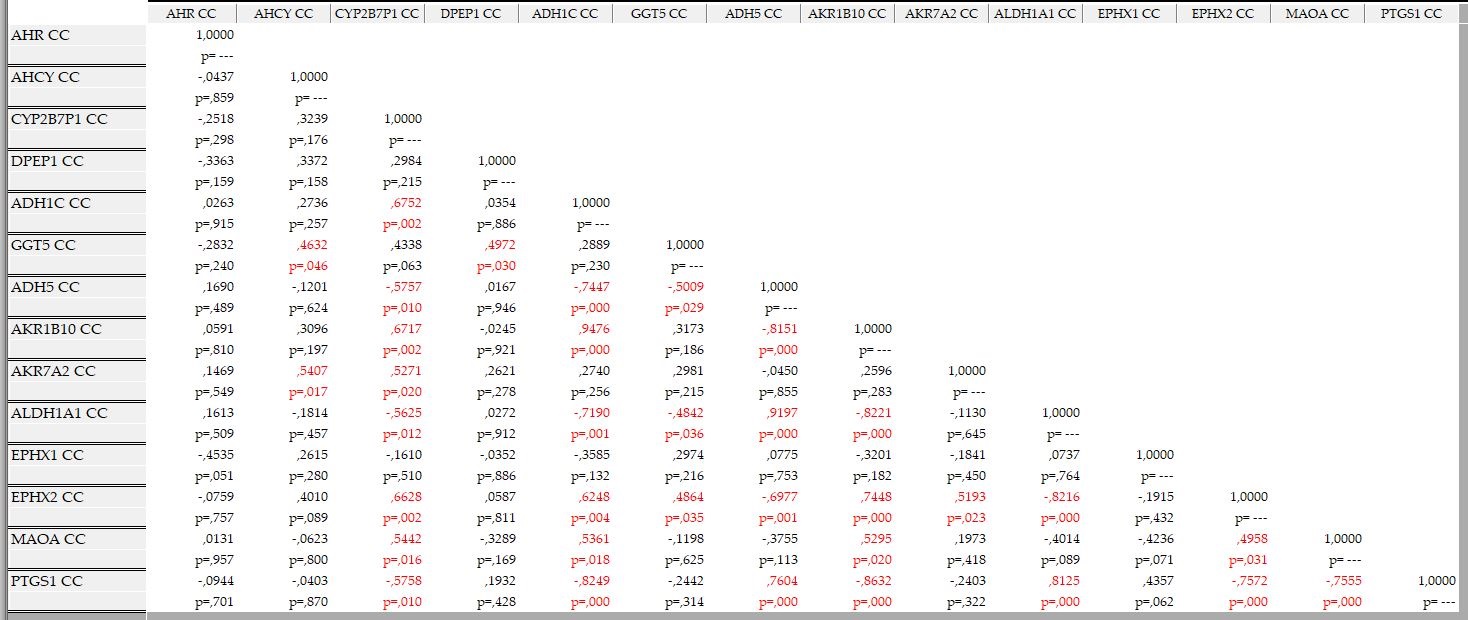

Supplement: Supplementary file 1 [file ijms-26-12116-s001.zip › Supplementary material/S19 Correlations of phase I transcripts distinguished in normal control large colon tissue (CC).jpg]

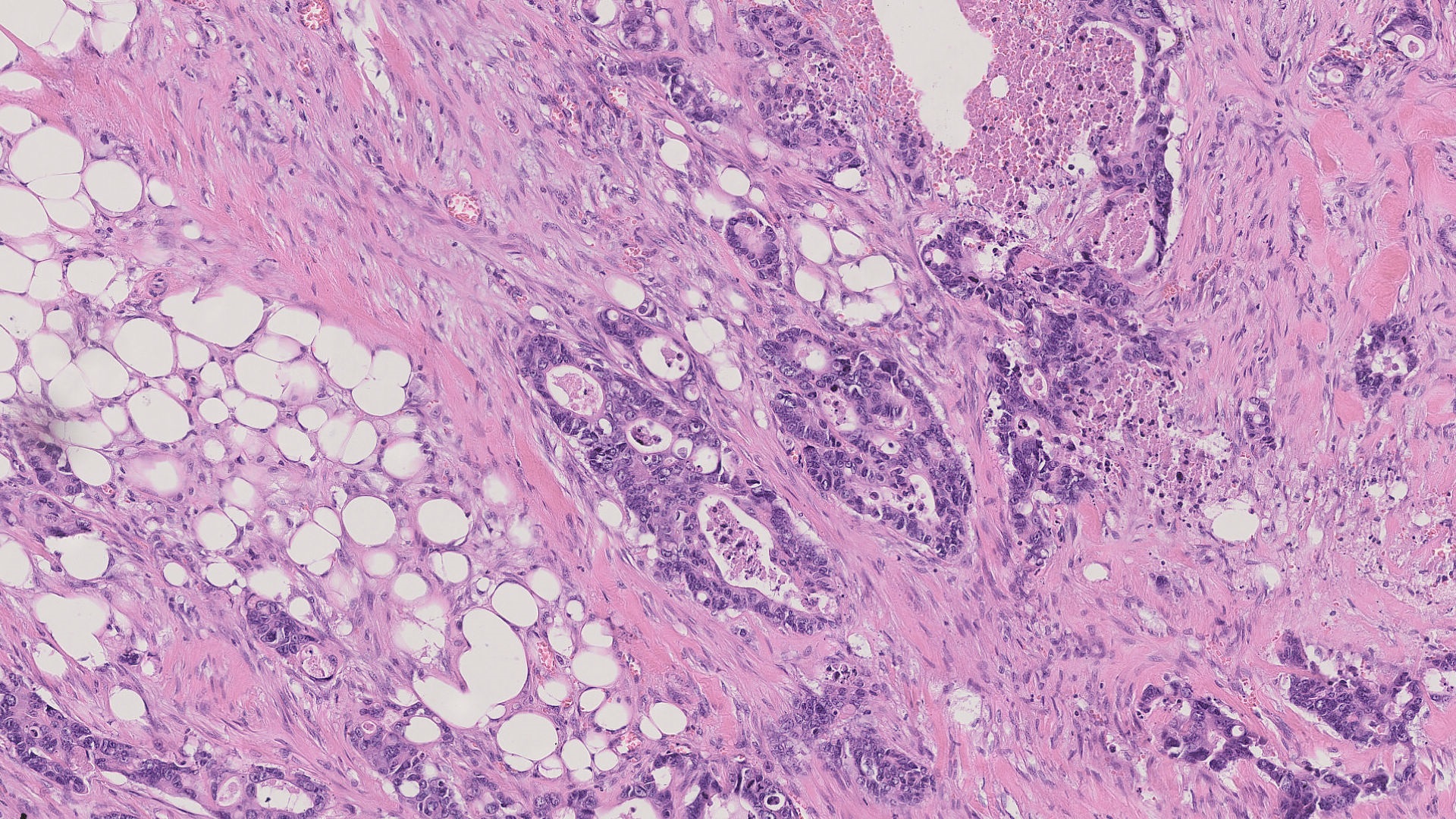

Supplement: Supplementary file 1 [file ijms-26-12116-s001.zip › Supplementary material/S2 Histopatological image of CRC in G2 (10x) primitive light, desmoplasm.jpg]

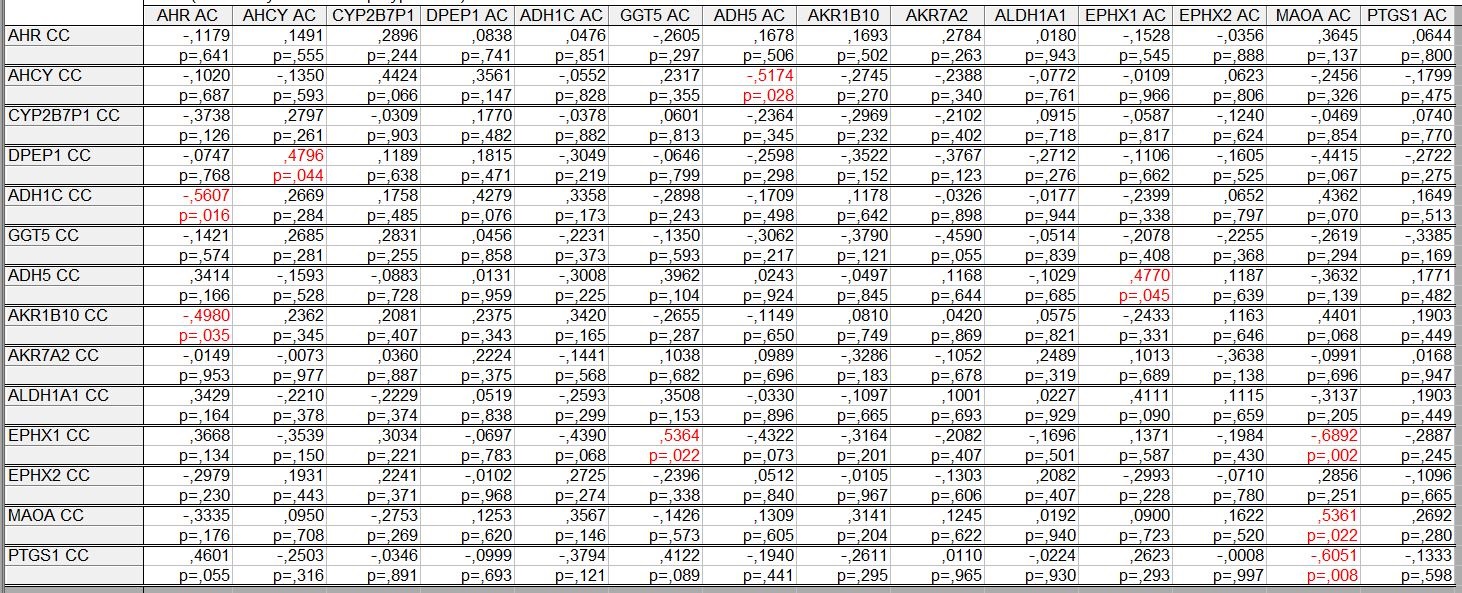

Supplement: Supplementary file 1 [file ijms-26-12116-s001.zip › Supplementary material/S20 Correlations of phase I transcripts distinguished in normal control large colon tissue (CC) CC vs in adenocarcinoma tissue (AC).jpg]

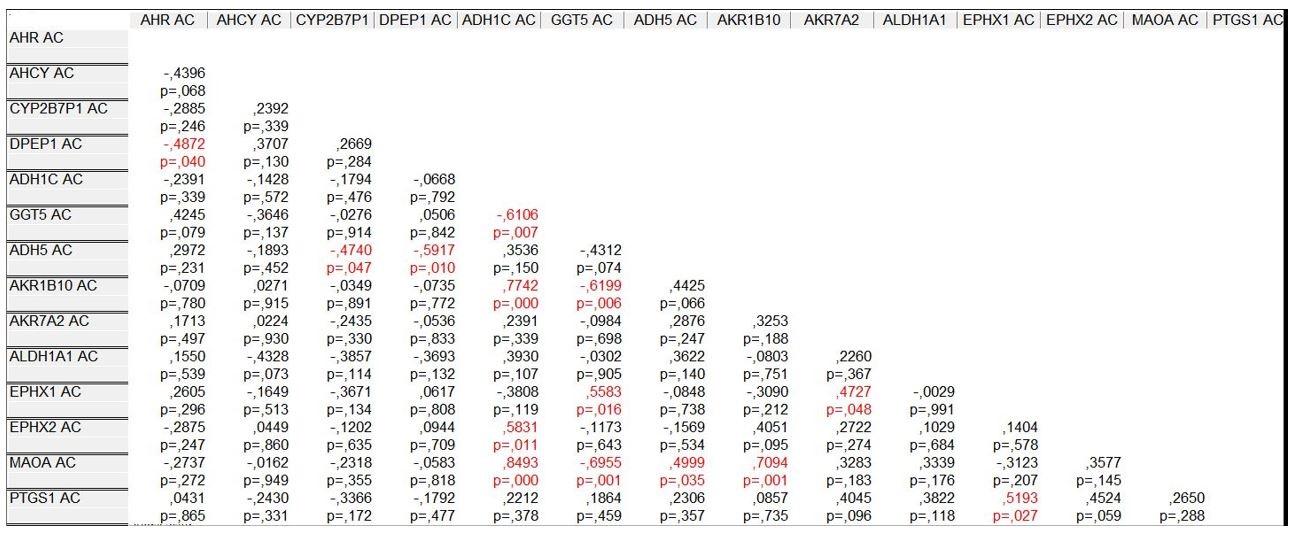

Supplement: Supplementary file 1 [file ijms-26-12116-s001.zip › Supplementary material/S21 Correlations of phase I transcripts distinguished in adenocarcinoma tissue (AC vs AC).jpg]

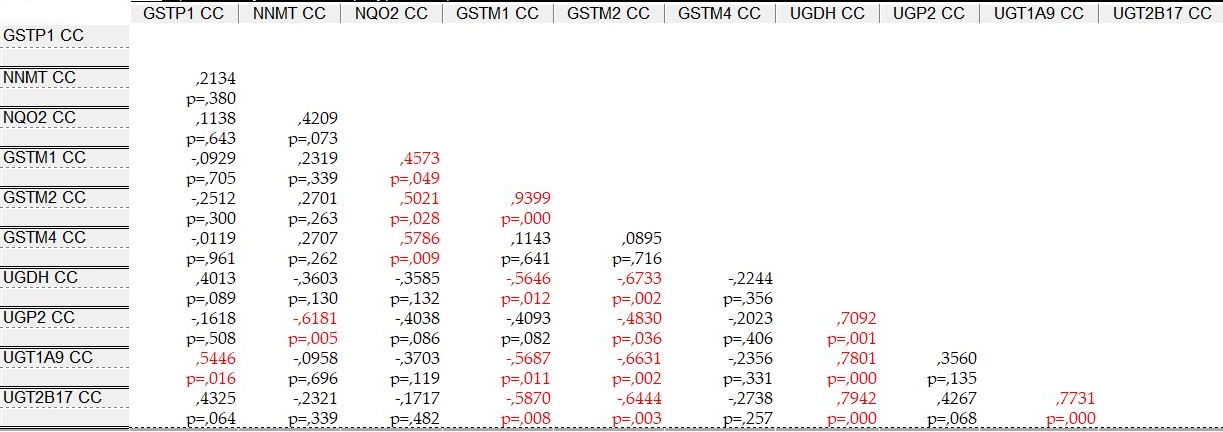

Supplement: Supplementary file 1 [file ijms-26-12116-s001.zip › Supplementary material/S22 Correlations of phase II transcripts distinguished in normal control large colon tissue (CC vs CC).jpg]

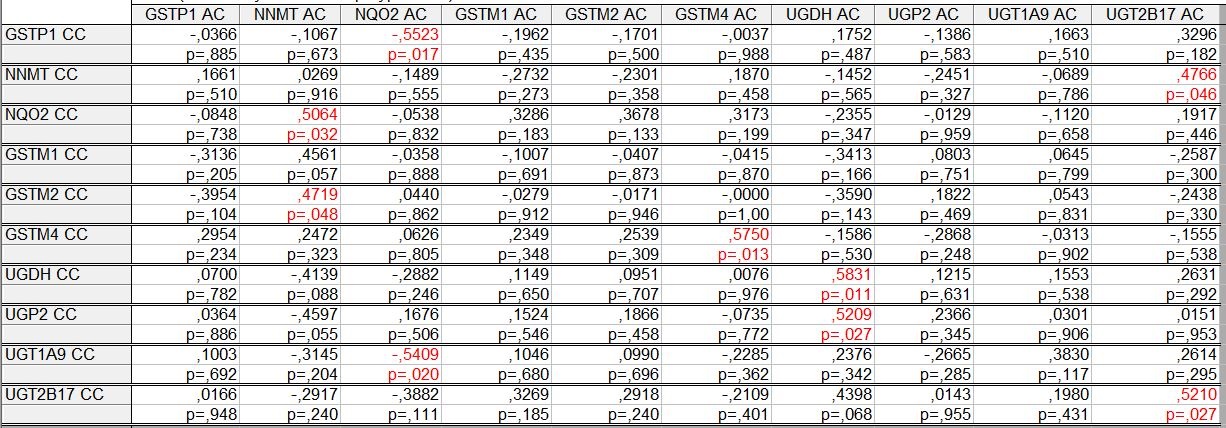

Supplement: Supplementary file 1 [file ijms-26-12116-s001.zip › Supplementary material/S23 Correlations of phase II transcripts distinguished in normal control large colon tissue (CC) CC vs in adenocarcinoma tissue (AC).jpg]

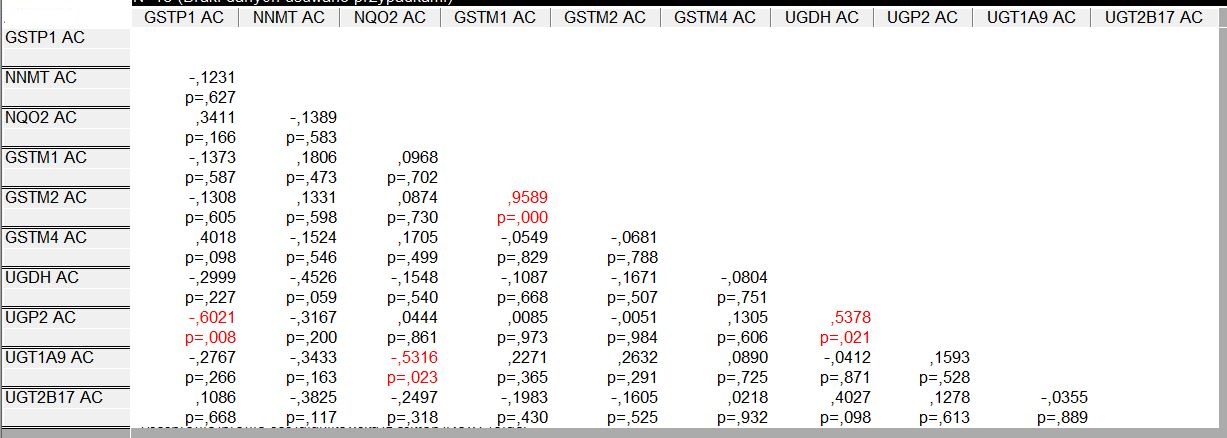

Supplement: Supplementary file 1 [file ijms-26-12116-s001.zip › Supplementary material/S24 Correlations of phase II transcripts distinguished in adenocarcinoma tissue (AC vs AC).jpg]

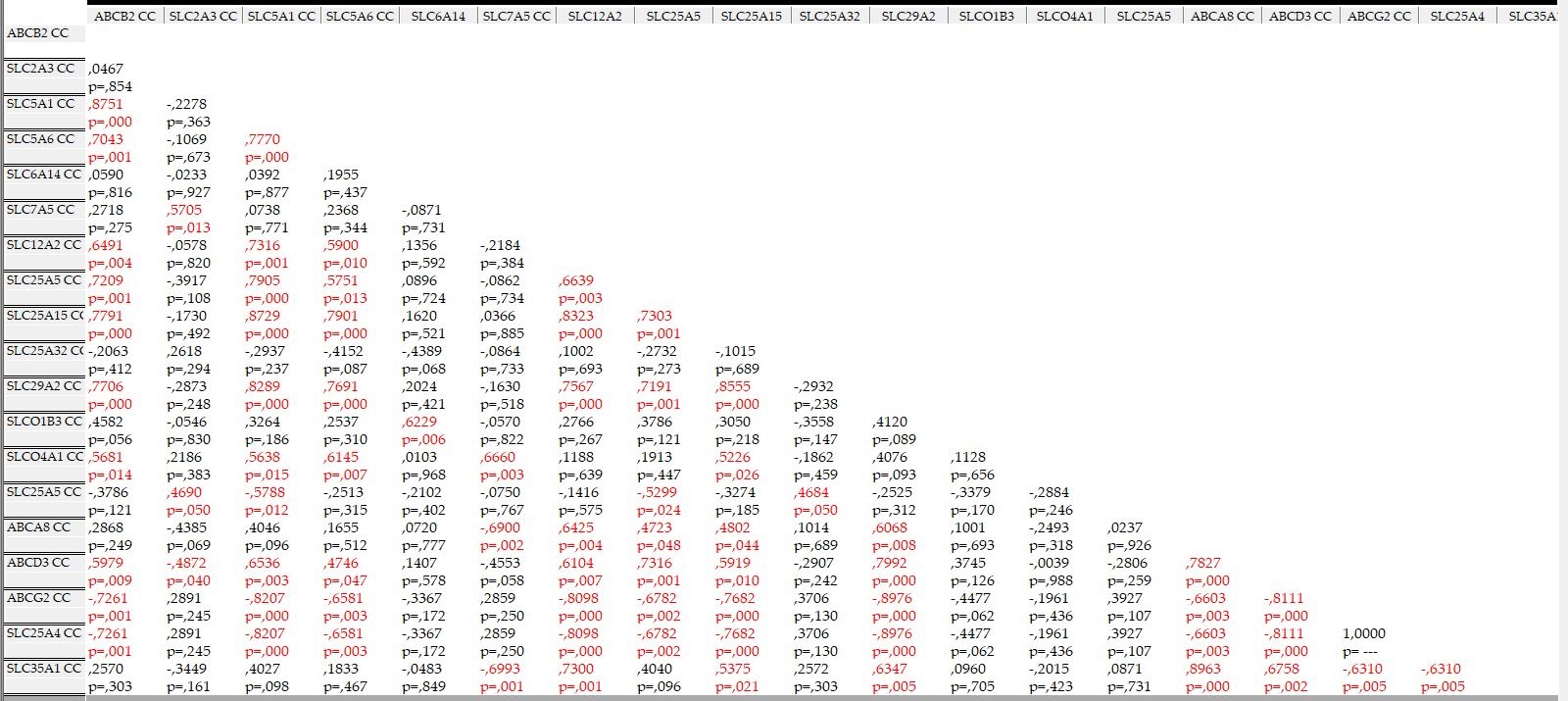

Supplement: Supplementary file 1 [file ijms-26-12116-s001.zip › Supplementary material/S25 Correlations of phase III transcripts distinguished in normal control large colon tissue (CC).jpg]

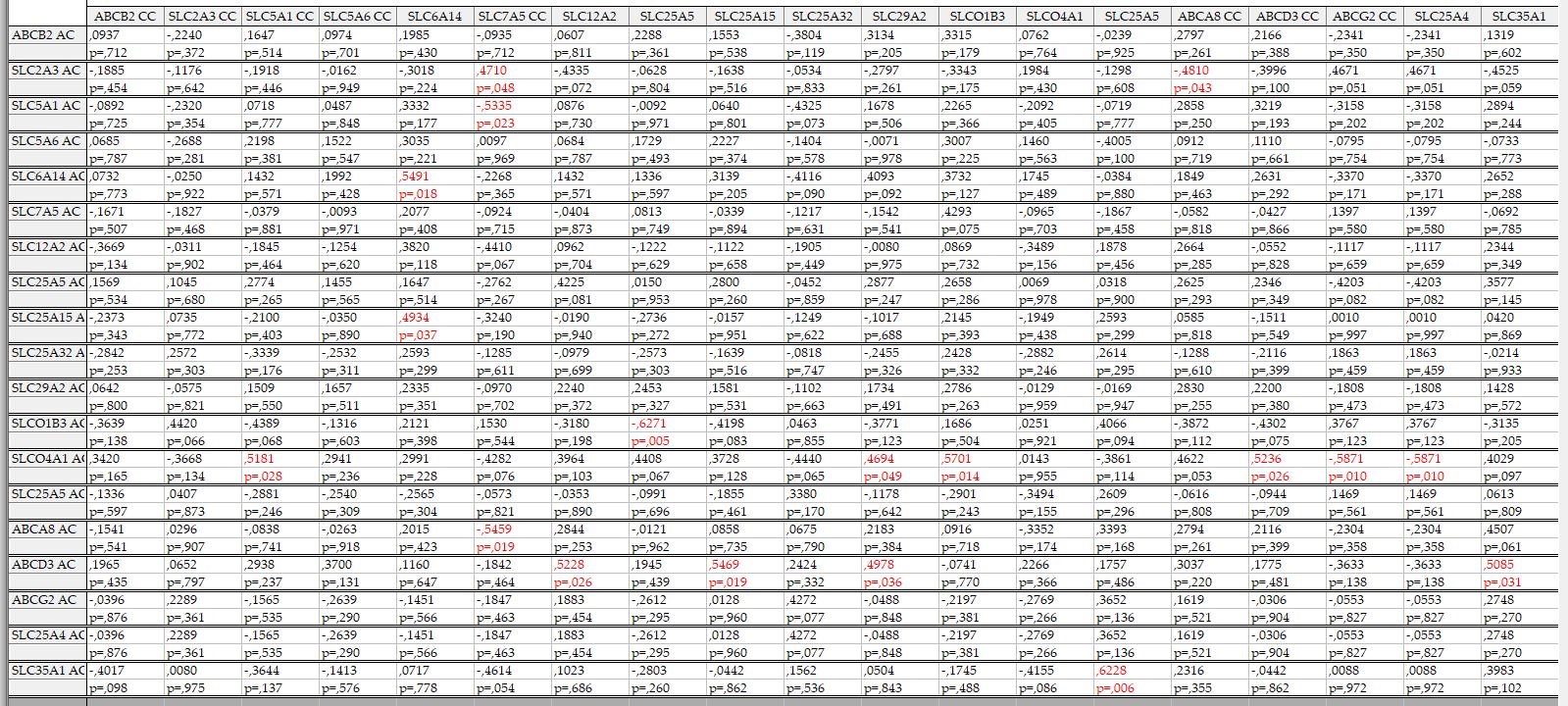

Supplement: Supplementary file 1 [file ijms-26-12116-s001.zip › Supplementary material/S26 Correlations of phase III transcripts distinguished in normal control large colon tissue (CC) CC vs in adenocarcinoma tissue (AC).jpg]

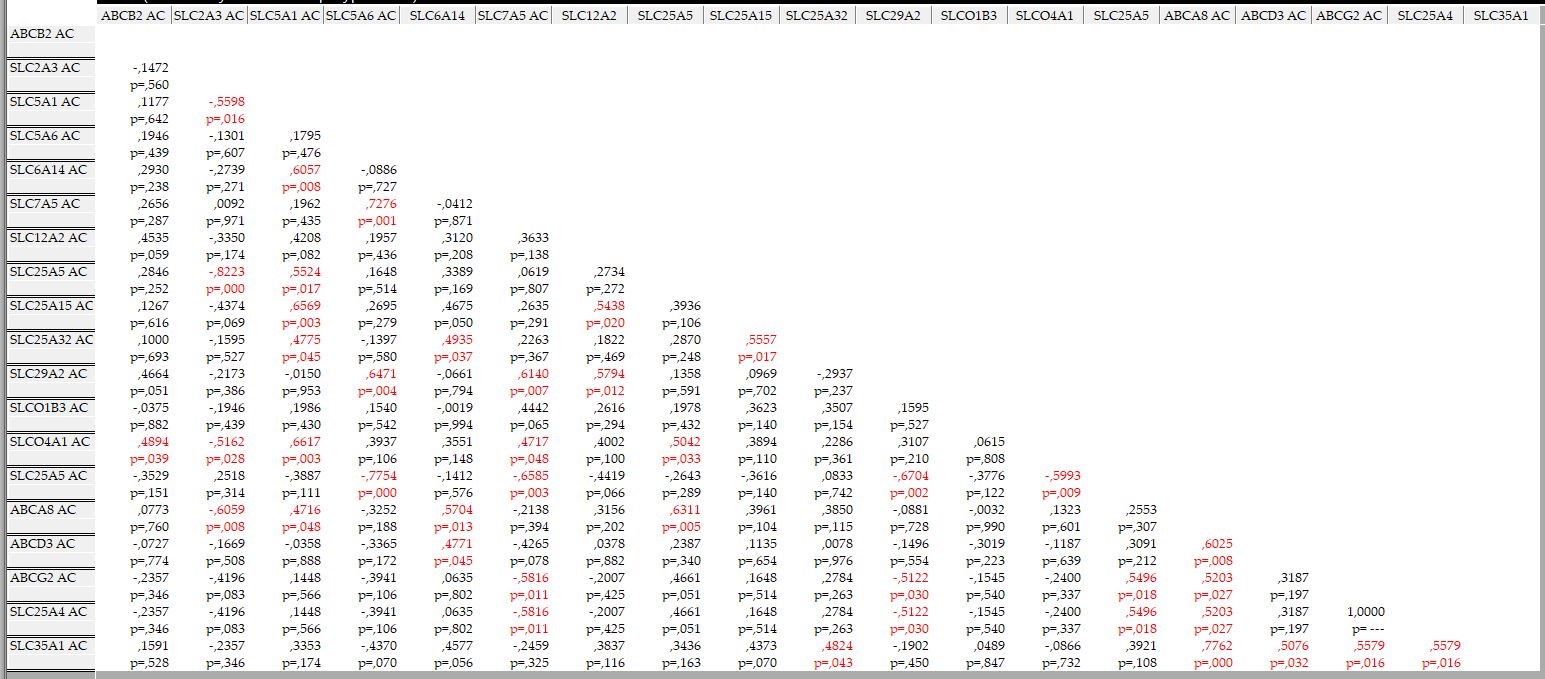

Supplement: Supplementary file 1 [file ijms-26-12116-s001.zip › Supplementary material/S27 Correlations of phase III transcripts distinguished in adenocarcinoma tissue (AC vs AC).jpg]

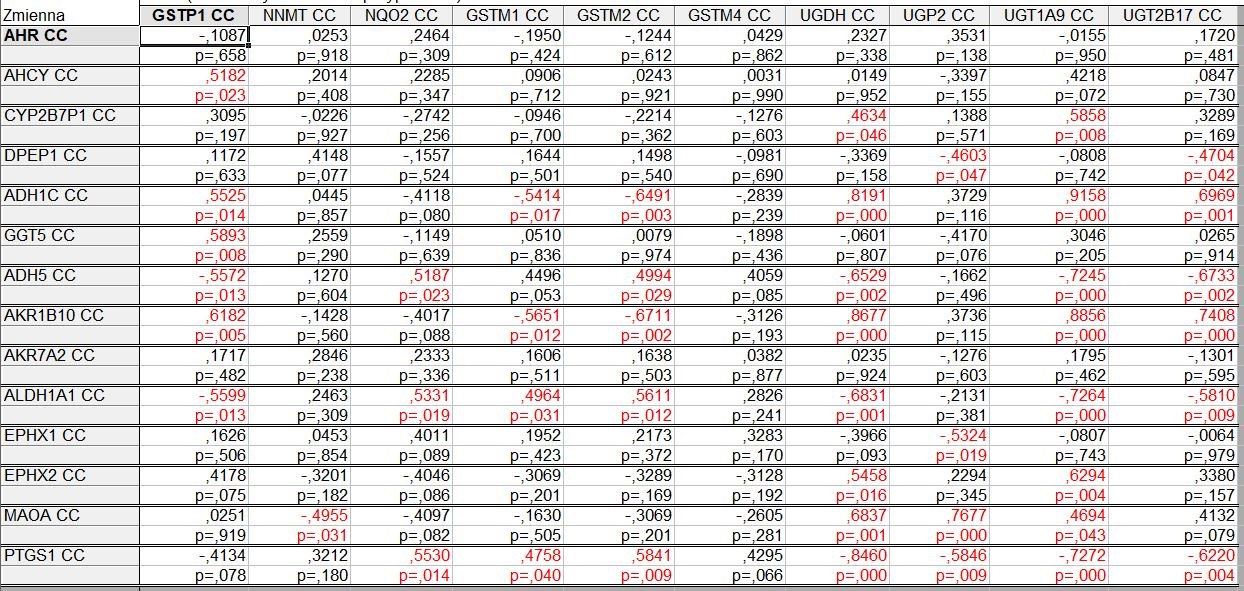

Supplement: Supplementary file 1 [file ijms-26-12116-s001.zip › Supplementary material/S28 Correlations of phase I and II transcripts distinguished in normal control large colon tissue (CC).jpg]

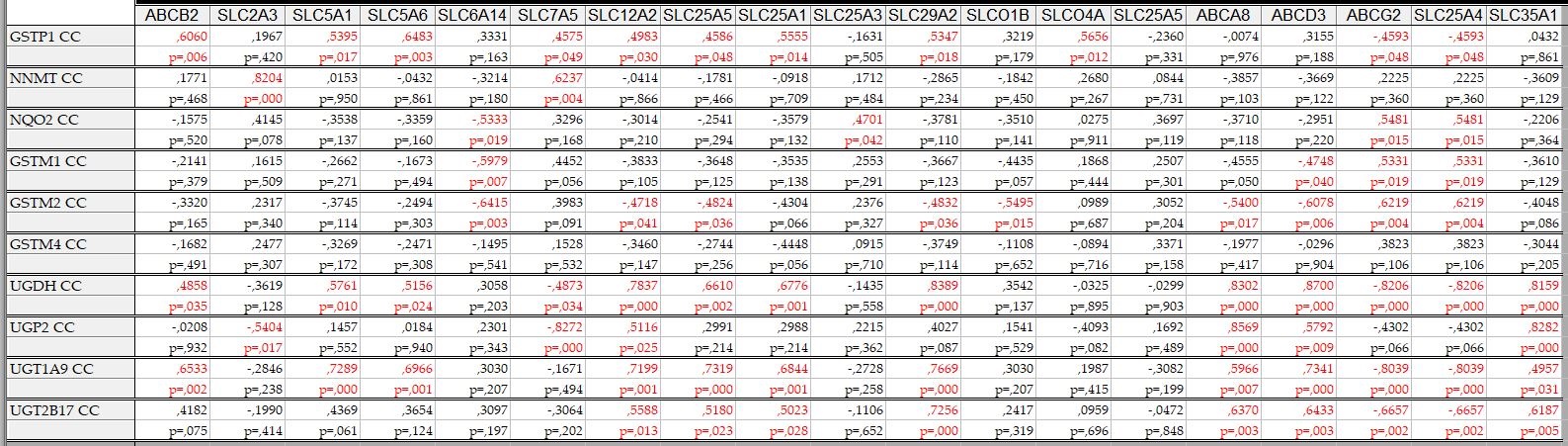

Supplement: Supplementary file 1 [file ijms-26-12116-s001.zip › Supplementary material/S29 Correlations of phase II and III transcripts distinguished in normal control large colon tissue (CC).jpg]

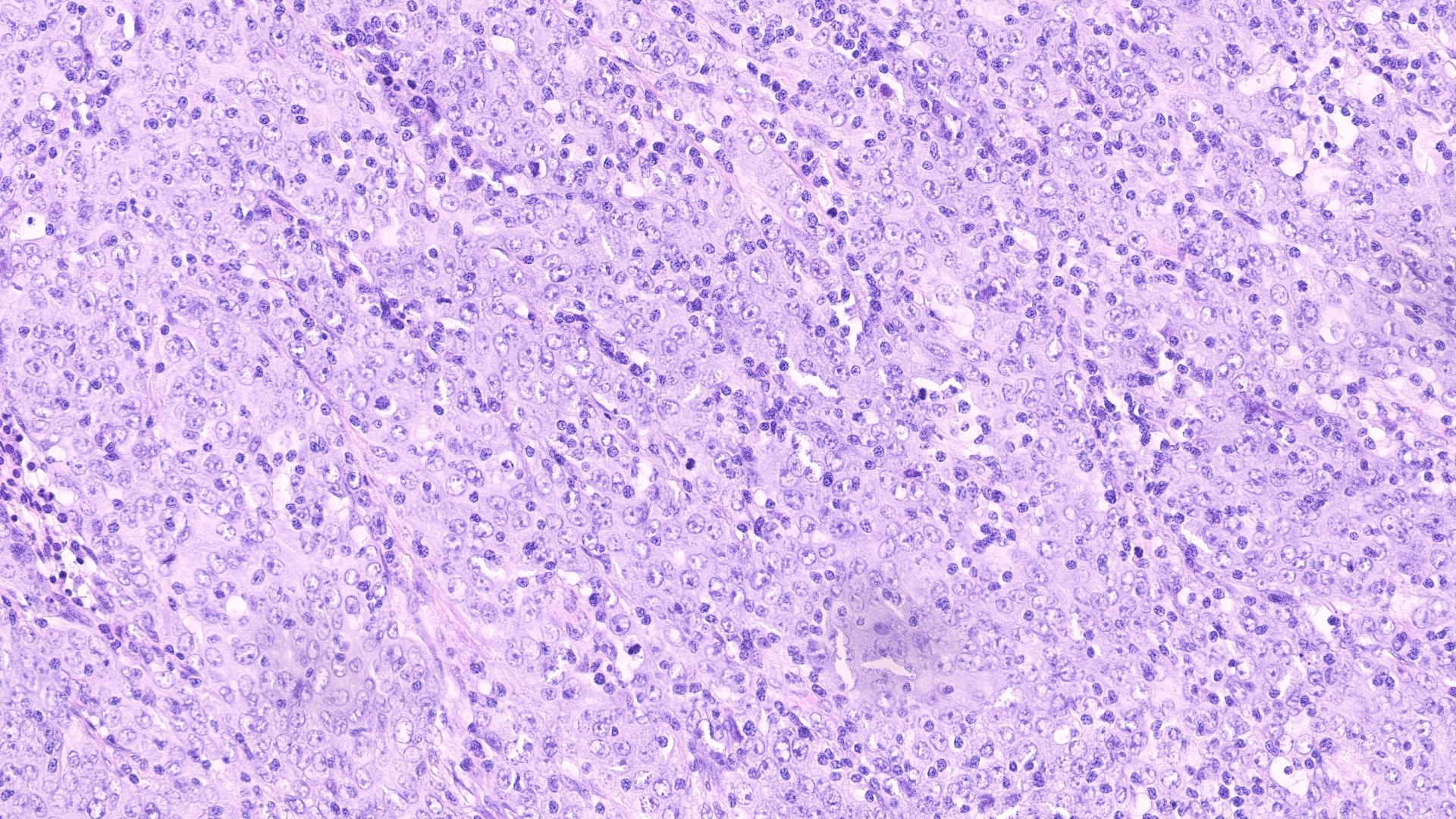

Supplement: Supplementary file 1 [file ijms-26-12116-s001.zip › Supplementary material/S3 Histopatological image of CRC in G3 (20x) primitive colon cancer cells.jpg]

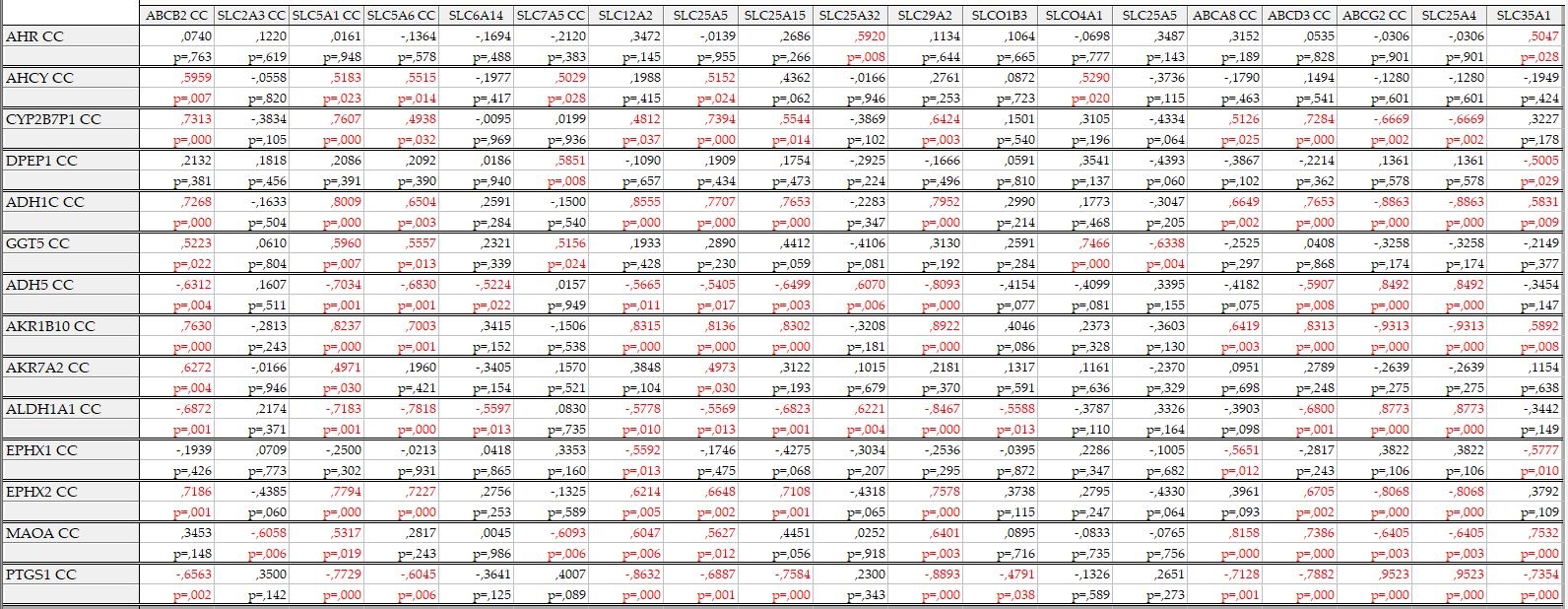

Supplement: Supplementary file 1 [file ijms-26-12116-s001.zip › Supplementary material/S30 Correlations of phase I and III transcripts distinguished in normal control large colon tissue (CC).jpg]

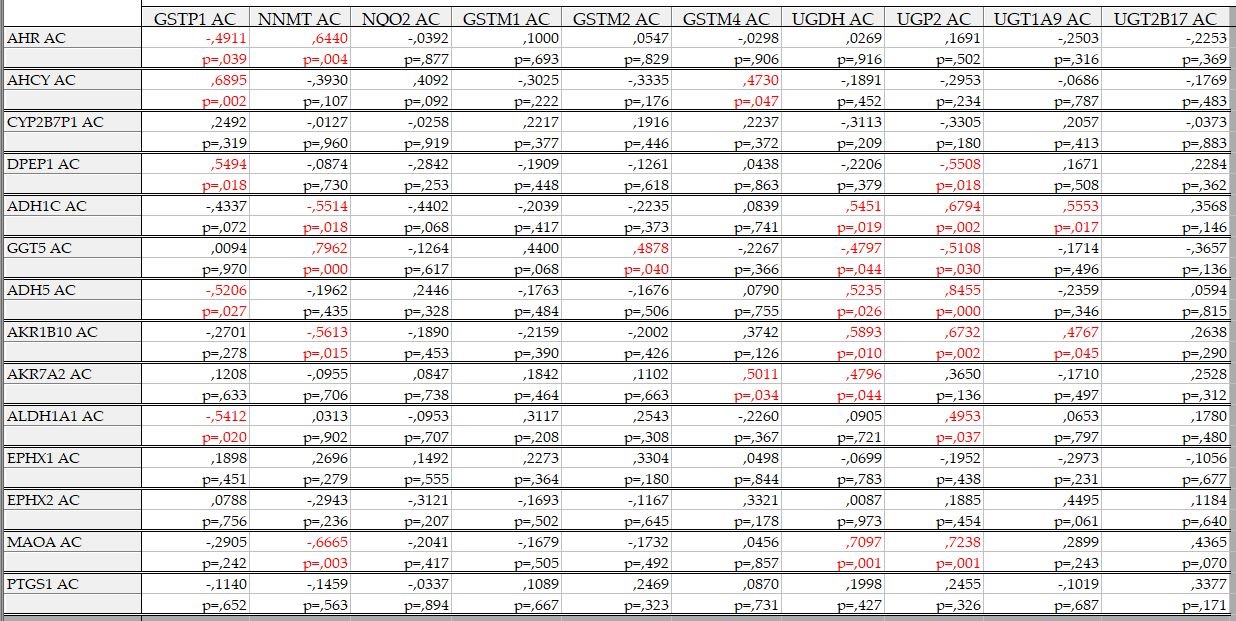

Supplement: Supplementary file 1 [file ijms-26-12116-s001.zip › Supplementary material/S31 Correlations of phase I and II transcripts distinguished in adenocarcinoma tissue (AC vs AC).jpg]

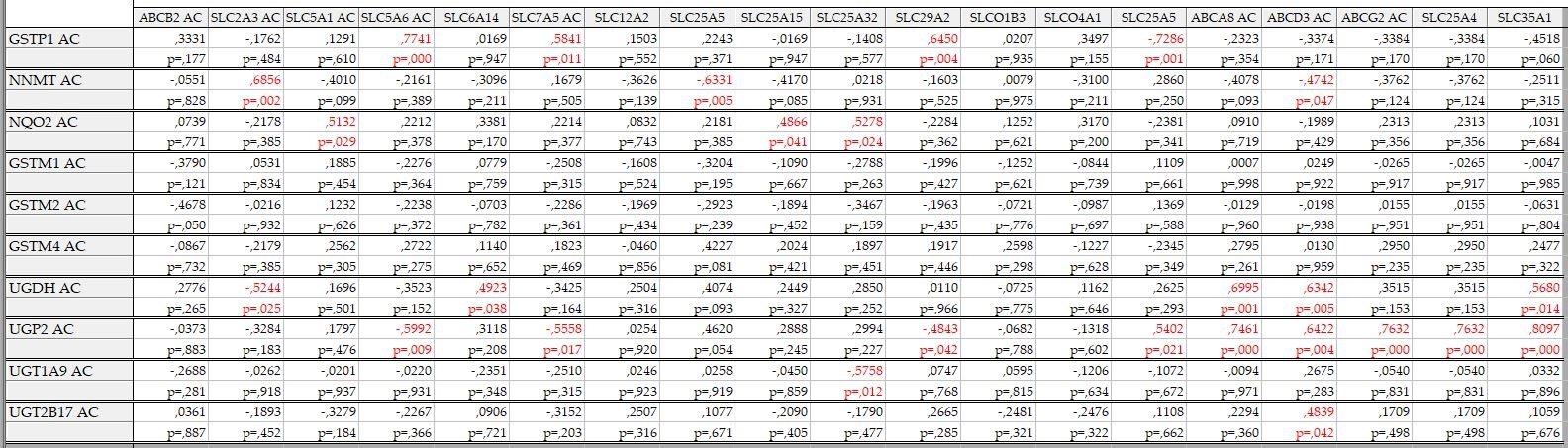

Supplement: Supplementary file 1 [file ijms-26-12116-s001.zip › Supplementary material/S32 Correlations of phase II and III transcripts distinguished in adenocarcinoma tissue (AC vs AC).jpg]

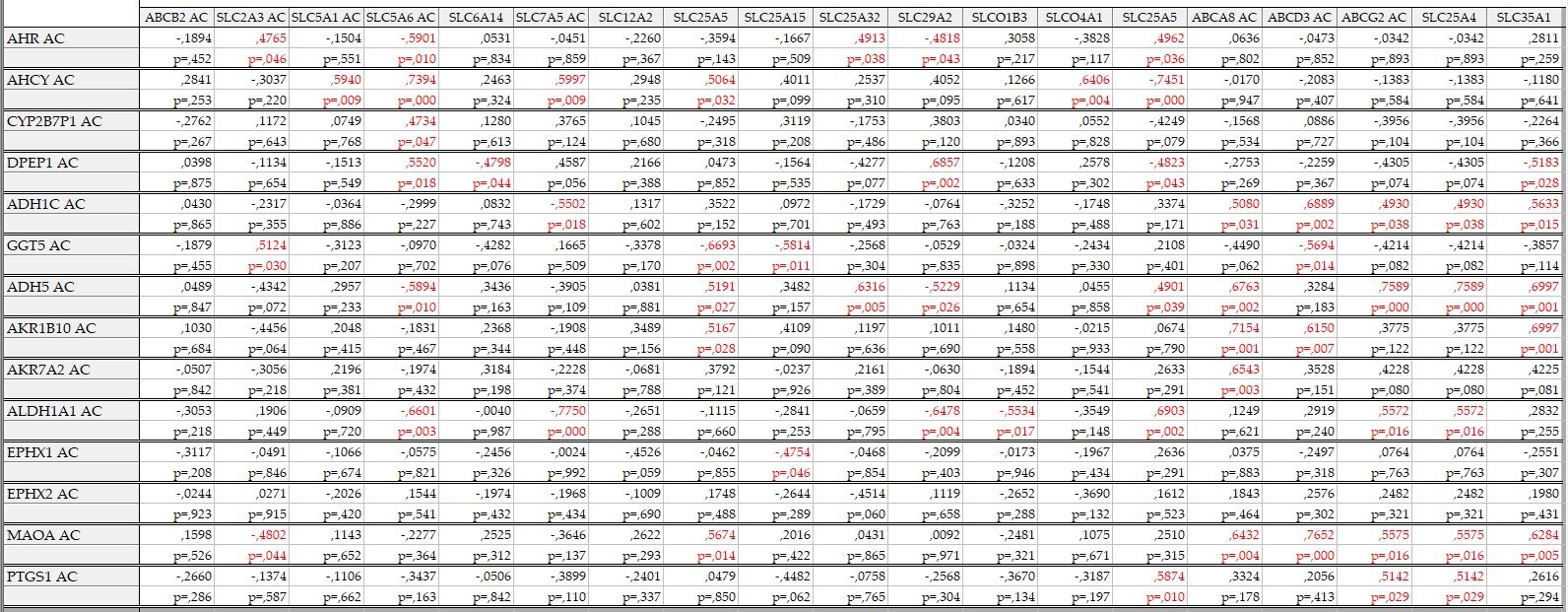

Supplement: Supplementary file 1 [file ijms-26-12116-s001.zip › Supplementary material/S33 Correlations of phase I and III transcripts distinguished in adenocarcinoma tissue (AC vs AC).jpg]

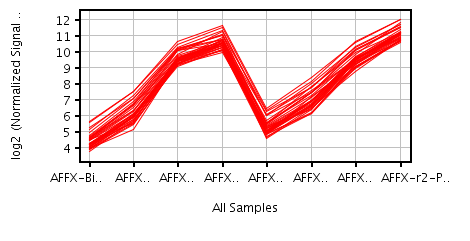

Supplement: Supplementary file 1 [file ijms-26-12116-s001.zip › Supplementary material/S4 Figure of Quality Control of CRC Microarrays.tiff]
